# Temporal variation of mycorrhization rates in a tree diversity experiment

**DOI:** 10.1002/ece3.10002

**Published:** 2023-04-19

**Authors:** Heike Heklau, Nicole Schindler, Nico Eisenhauer, Olga Ferlian, Helge Bruelheide

**Affiliations:** ^1^ Institute of Biology/Geobotany and Botanical Garden Martin Luther University Halle‐Wittenberg Am Kirchtor 1 Halle (Saale) 06108 Germany; ^2^ German Centre for Integrative Biodiversity Research (iDiv) Halle‐Jena‐Leipzig Puschstr. 4 Leipzig 04103 Germany; ^3^ Institute of Biology Leipzig University Puschstr. 4 Leipzig 04103 Germany

**Keywords:** Arbuscular mycorrhiza, biodiversity–ecosystem functioning experiment, deciduous trees, dual mycorrhization, ectomycorrhiza, temporal variation of mycorrhiza rates

## Abstract

While mycorrhization rates have been studied in different contexts, not much is known about their temporal patterns across seasons. Here, we asked how mycorrhization rates of 10 deciduous trees assessed by microscopy changed from winter to spring to early summer. We made use of a tree diversity experiment on nutrient‐rich soil (former farmland) in Central Germany. In the experiment, saplings of host species with a preference for either arbuscular mycorrhiza (AM) or ectomycorrhiza (EM) were planted in monocultures, two‐species, and four‐species mixtures. In addition, mixtures were composed of tree species of only one mycorrhizal type or by AM/EM trees. For almost all species, with the exception of *Aesculus hippocastanum* and *Acer pseudoplatanus* (only AM), dual mycorrhization with both types (AM and EM) was found at every sampling time (December, March, and May), although the expected preferences for certain mycorrhizal types were confirmed. The sampling date had a significant influence on mycorrhization rates of both EM and AM tree species. Frequencies of EM and AM were lowest in May, but there were no differences between December and March. The causes of this seasonal variation may be associated with climate‐induced differences in carbon allocation to mycorrhizal tree roots in the temperate climate. Within individual trees, mycorrhization rates by AM and EM fungi were not correlated over time, pointing to asynchronous variation between both types and to independent drivers for AM and EM mycorrhization. At the community level, mycorrhiza frequency of either of the two types became more asynchronous from two‐species to four‐species mixtures. Thus, increased community asynchrony in mycorrhization could be another important mechanism in biodiversity–ecosystem functioning relationships.

## Graphical abstract

The sampling date had a significant influence on mycorrhization rates, of both EM and AM tree species. Frequencies of EM and AM were lowest in May, but there were no differences between December and March. The causes of this temporal variation must be associated with climate‐induced differences in carbon allocation to mycorrhizal tree roots in the temperate climate.

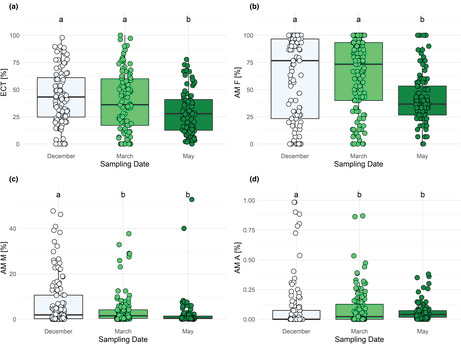

## 1 INTRODUCTION

Mycorrhizal fungi are directly involved in numerous soil ecological processes (Kaiser et al., 2010; Koranda et al., 2013) and indirectly also in many aboveground processes (review of knowledge in Tedersoo et al., 2020). While the temporal variation of these processes has been studied in detail (Hansen & Beck, 1994; Kuptz et al., 2011), not much is known about the factors that influence the seasonal variation in mycorrhizal colonization rates. Most data on seasonal variation come from indirect evidence, such as from soil respiration. For temperate and boreal trees, which mostly form associations with ectomycorrhiza (EM), Epron et al. (2011, 2012) reported that the amount of carbon transferred to their fungal partners varies with season, in particular responding to the variation in temperature. This seasonal pattern of soil respiration seems to follow more closely the belowground carbon allocation of the trees rather than the seasonal course of soil temperature, as was demonstrated for a boreal pine forest by Högberg et al. (2001). However, the EM‐forming species of Basidiomycetes or Ascomycetes do not depend exclusively on the carbon supply from the host as they have a limited ability to also utilize lignin and cellulose (Smith & Read, 2009, p. 300). It is known that ectomycorrhizal fungi are able to switch from symbiosis to saprotrophy at low carbon supply from their hosts (Kaiser et al., 2010). However, also the degradation of organic matter by ectomycorrhizal fungi follows a seasonal pattern. While cellulase and protease activities were found to be highest in November, phenoloxidase and peroxidase activities peaked in September (Kaiser et al., 2010). In contrast, fungal species of Glomeromycota, which form arbuscular mycorrhiza (AM), derive the total amount of carbon from their hosts (Willis et al., 2013), making them obligate symbionts. These differences in the degree of dependence on carbon supply from host plants suggest that the EM and AM fungi might also differ in their seasonal activity.

Based on literature data, a comparison of EM and AM fungi with respect to their seasonal activity is hampered by the fact that these two groups are usually studied in different ecosystems. In temperate forests, the activity of both types of fungal associations seems to peak in summer. For example, the relative amount of ^13^C the EM tree *Fagus sylvatica* allocated to belowground microbial respiration was only 3% of ^13^C in soil CO_2_ efflux in May, rose to a maximum of 18%–21% in July, and fell again to 1%–6% in August (Epron et al., 2011). The authors explain this pattern with storage remobilization for leafing out in spring (April–May). During this time of increased need for carbohydrates for growth, the host plants reduce the carbon release to the rhizosphere, which also happens at the end of the vegetation period in autumn. A pronounced similar seasonal pattern of carbon allocation was also reported for the AM trees of the *Populus* genus (Horwath et al., 1994; Mikan et al., 2000). The respired proportion of ^14^C‐labeled carbon translocated to root system of *P. euroamericana* amounted to 45% in July and 24% in September (Horwath et al., 1994). Similarly, for *P. tremuloides*, Mikan et al. (2000) described that maximum rates of soil respiration occurred from mid to late June and minimum rates in early October. Unfortunately, to our knowledge, there is no study site where seasonal activities were studied simultaneously on EM and AM tree species.

It should be expected that such fluctuations in assimilate supply from the host trees to AM fungi or changes in the lifestyle of EM fungi are also reflected in mycorrhization rates, which are the morphological manifestation of the symbiosis. While AM mycorrhization rates can be quantified as the frequency of intracellular hyphae, arbuscules or vesicles (McGonigle & Fitter, 1990; Sun & Tang, 2012; Toth, 1992; Trouvelot et al., 1986; Vierheilig et al., 1998), EM mycorrhization rates can be assessed as frequency of root tips with a hyphae mantle around the root and a Hartig net, formed by hyphae between cortex cells (Brundrett & Tedersoo, 2020). Indeed, mycorrhization rates were found to indicate seasonal patterns. For tropical herb species in Bangladesh, Halder et al. (2015) demonstrated such a seasonal variation, with higher AM colonization rates in the rainy than in the dry season. Similarly, so Fakhech et al. (2019) and Meddad‐Hamza et al. (2017) described seasonal differences in mycorrhization rates for AM shrubs and trees between moist winter and dry summer in the Mediterranean area. However, not much is known about the seasonality of mycorrhization rates in temperate tree species.

Mycorrhization has various aspects that might vary differently with seasonality. AM is characterized mostly by both arbuscules and vesicles in the root cortex. The presence of arbuscules unequivocally indicates the nutrient transfer between host and fungus (Rich et al., 2021). Trouvelot et al. (1986) distinguished between the frequency of arbuscular mycorrhiza (AM F), which is the proportion of the number of root fragments that are colonized by AM fungi (arbuscules or vesicles or both present), the intensity of the arbuscular mycorrhizal colonization (AM M), which is based on the proportion of the root tissue colonized with vesicles as well as arbuscules, and finally the relative abundance of arbuscules (AM A). These indicators of AM mycorrhization rates might vary independently from each other. For example, along an elevational gradient of increasing precipitation and decreasing temperature in Tibet, Gai et al. (2012) found that only the intensity of colonization (AM M) in herbaceous plants decreased consistently with elevation, while the frequency of colonization and relative abundance of arbuscules varied more idiosyncratically.

Similarly, EM associations show temporal variation. Blasius et al. (1989) studied mycorrhization with EM in *P. abies* of Central Europe over 3 years and reported mycorrhization rates that varied with the growing season. Coll et al. (2012) analyzed EM of the evergreen *Q. ilex* and the deciduous *Q. faginea* in north‐eastern Spain. In both species, the highest percentage of non‐ectomycorrhizal fine roots was encountered in the dry summer (20%–30%) and the lowest one in the moist winter (5%–10%), which indicates a lower carbon supply for the fungi under drought conditions. Conclusions on the potential drivers of seasonal fluctuation in mycorrhization rates can also be drawn from inter‐annual comparisons. Collado et al. (2019) related data on EM mushroom yield (biomass and density) to tree‐ring growth in Europe. A correlation was only observed in Mediterranean forests, not in temperate or boreal ones, pointing to a strong effect of precipitation on both, mycorrhization (here better fruit body development) and carbon allocation, while there was only a weak correlation between mycorrhization and carbon allocation under favorable conditions.

If both fungi of AM and EM of deciduous trees equally depend on carbon allocation from the host plant, one would expect that both types should respond similar to seasons, resulting in synchrony, which should be seen in a correlation in mycorrhization rates between both types over time. However, there is also evidence that the environmental drivers for AM‐ and EM‐dominated ecosystems are of the utmost significance. In a worldwide synthesis, Vargas et al. (2010) showed that the seasonal patterns of ecosystem CO_2_ fluxes in EM‐dominated woody vegetation types were primarily controlled by changes in mean annual temperature, whereas those in AM‐dominated vegetation types were driven by changes in precipitation. This ultimately should result in asynchronous patterns in these two types of mycorrhization.

There is also some circumstantial evidence for asynchrony in mycorrhization rates between ectomycorrhiza (EM) and vesicular–arbuscular mycorrhiza (AM), which comes from dual‐mycorrhizal plant species. These species can form both EM and AM symbioses (Heklau et al., 2021; Teste et al., 2020). In dual‐mycorrhizal plants, the mycorrhizal type dominance can vary with the life‐history stage (seedling versus adult plant). Chen et al. (2000) found that EM is, in fact, more important than AM in adult *Eucalyptus* trees, while AM can provide benefits during seedling establishment. For the dual‐mycorrhizal species *Quercus agrifolia* in California, Querejeta et al. (2009) found that the colonization with EM fungi and soil hyphal density were strongly correlated with moisture across sites and year, while AM fungi colonization was not. During severe droughts, *Q. agrifolia* exclusively formed AM. In plants with dual associations, AM might be relictual (Brundrett, 2002) and could be an insurance strategy (Teste et al., 2020). However, in dual‐mycorrhizal plant species, synchrony between EM and AM might be affected by mycorrhizal‐spillover effects from adjacent host species (Eagar et al., 2022). Such a mycorrhizal spillover was described by Dickie et al. (2001) when the authors found high AM colonization rates of *Q. rubra* seedlings if they grew away from EM *Quercus* trees, while these were low if the seedlings grew near EM *Quercus* trees. According to these findings, trees might be able to intensify their own overall state of mycorrhization by forming an additional symbiosis with the contrasting mycorrhiza type of an adjacent host species. For the same experiment as in the current study, Heklau et al. (2021) described that mixing of AM‐ and EM‐associated tree hosts affected the tree mycorrhization rates and the hosts' fungal community composition. Although it is well known that mycorrhizal fungi form a link between different host species and form belowground networks (Bahram et al., 2011; Weiss et al., 2004), it remains unknown how this network dampens or intensifies synchrony. For example, synchrony might depend on the number of different interaction partners that are part of the symbiosis. Such questions can only be studied in biodiversity–ecosystem functioning (BEF) experiments, where host tree richness has been manipulated, thus avoiding confounding with the multitude of factors that drive mycorrhization in natural forest ecosystems.

Here, we studied the seasonality of mycorrhization rates in AM‐ and EM‐associated trees, making use of the MyDiv BEF experiment in Central Germany (Ferlian et al., 2018). We hypothesized that the mycorrhization rates of both AM and EM (1) depend on time of sampling and that (2) differed in their temporal courses, with EM being negatively affected by warm and dry weather conditions. Furthermore, we tested the hypothesis that (3) such opposing trends of AM and EM are caused by an inverse correlation between AM and EM mycorrhization rates within single trees. This would happen if each single tree switched mycorrhization types according to the particular environmental conditions in the respective season. Alternatively, or in combination, opposing trends of AM and EM mycorrhization might occur among species in a tree community, causing asynchrony at the community level. As the trees in the MyDiv experiment were planted in monocultures, two‐species, and four‐species mixtures, and in addition, the mixtures were composed of tree species of only one mycorrhizal type (EM or AM) or by mixtures of AM and EM trees, we finally hypothesized that (4) synchrony among different tree species in the community depend on the type of mixtures and tree richness levels.

## 2 MATERIALS AND METHODS

### 2.1 Site

The experimental site is located in Saxony‐Anhalt, Central Germany, southwest of Halle (51°23′ N 11°53′ E) at 114–116 m a. s. l. at the Bad Lauchstädt Experimental Research Station of the Helmholtz Centre for Environmental Research–UFZ (Ferlian et al., 2018). Table 1 provides weather data at the time of sampling (December 2017, March, and May 2018) and long‐term climatic conditions. Weather in December 2017 was wet and cold, with only moderate frosts, while March 2018 showed the lowest temperatures. Subsequently, May 2018 was extraordinarily dry and very warm. The soil is a Haplic Chernozem (Altermann et al., 2005). Until 2012, this site had been used for agriculture at which point it was converted to grassland for 2 years before it was plowed to prepare the site for planting the trees in March 2015 (Ferlian et al., 2018).

**TABLE 1:** Weather data at the time of sampling (December 2017, March, and May 2018) and long‐term averages (from 1956 to date) at the Bad Lauchstädt Experimental Research Station of the Helmholtz Centre for Environmental Research–UFZ with a mean annual temperature of 8.8°C and a mean annual precipitation of 484 mm.

Climate measurements	December 2017	March 2018	May 2018
Start date of sampling	December 1, 2017	March 1, 2018	May 1, 2018
End date of sampling	December 31, 2017	March 31, 2018	May 31, 2018
Monthly average of air temperature [°C]	3.55	2.17	16.51
Monthly average of maximum air temperature [°C]	5.87	6.54	22.49
Monthly average of minimum air temperature [°C]	0.81	−2.4	9.41
Absolute maximum of air temperature [°C]	13.5	17.4	31.3
Absolute minimum of air temperature [°C]	−5.7	−12.2	1.2
Monthly sum of sunshine duration [h]	45.18	132.72	311.12
Monthly sum of precipitation [mm]	20.6	41.9	13.2
Maximum of daily precipitation in the sampling month [mm]	3.6	11.2	5.7

### 2.2 Experimental design

In the experiment, One hundred and forty 2‐ to 3‐year‐old tree individuals were planted per plot at a distance of 1 m in 2015, on a total of 80 plots (plot size 11 × 11 m) organized in two blocks (for details see Ferlian et al., 2018). Ten tree species were included in this experiment, each five of them predominantly associated with either AM or EM (in the following called Myc_Type and referring to the preferred mycorrhizal association of the host species). The predominant mycorrhiza type (EM or AM) of each sample tree species was assessed according to the relevant literature (see Ferlian et al., 2018; Heklau et al., 2021). While *Acer pseudoplatanus* L.*, Aesculus hippocastanum* L., *Fraxinus excelsior* L.*, Prunus avium* L., and *Sorbus aucuparia* L. were reported to be associated predominantly with AM fungi, *Betula pendula* Roth*, Carpinus betulus* L., *Fagus sylvatica* L.*, Quercus petraea* (Matt.) Liebl., and *Tilia platyphyllos* Scop. are thought to preferentially form symbioses with EM fungi. Trees of these two main mycorrhizal types were planted in monocultures, two‐species, and four‐species mixtures (in the following abbreviated with Mono, Di, and Tetra mixtures), which together formed a tree species richness gradient (Spec_Rich, tree species richness). Additionally, the two‐species and four‐species mixtures contained only AM trees, only EM trees, or AM and EM trees in mixture. Thus, each plot had two states of mycorrhiza mixture type (in the following called Mix_Type and referring to the preferred mycorrhizal associations that occurred in a plot), either monotypic (AM or EM) or mixed (AM and EM trees growing together). For this study, 36 out of 80 plots were selected and the root samples were taken from two trees of each species in the southwest corner of selected plots (Figure S1).

### 2.3 Sampling

Fine roots of all tree species were sampled from 0 to 20 cm soil depth, using a spade to expose the root system. We selected numerous fine roots close to the stem and made certain that the roots belonged to the target tree. Sampling took place in December 2017 (5th, 7th, 8th), March 2018 (9th, 10th, 13th), and May 2018 (10th, 11th). In total, we took 124 root samples at each sampling time, 20 samples each of *A. pseudoplatanus, C. betulus, F. sylvatica*, and *P. avium*, and eight each of *B. pendula, S. aucuparia*, and *T. platyphyllos*, seven each of *A. hippocastanum* and *F. excelsior*, and six of *Q. petraea*, altogether 372 root samples. The unequal sample sizes were the result of focusing upon *A. pseudoplatanus, C. betulus, F. sylvatica*, and *P. avium*. In the laboratory, fine roots were washed gently. The morphological analysis of the ectomycorrhiza was carried out on fresh material on subsequent days. For analysis of the AM, additional fine roots of each root sample were placed in ethanol (50%) and kept cool in the refrigerator at 4°C for several weeks.

### 2.4 Morphological preparation of root samples

Root tips were examined using a dissecting microscope (Stemi DV 4; Zeiss, Jena, Germany). From each tree individual, three 5‐cm root pieces of the first order were assessed as colonized with ectomycorrhiza, as indicated by a lighter color and swollen tips, or as not colonized (Figures [Link], [Link]). For further analysis, the frequency of active ectomycorrhizal root tips (ECT) was calculated using the following formula (1):

(1) $$\mathrm{ECT}=\frac{\text{Number of root tips with ectomycorrhiza}}{\text{Number of total root tips examined}}\times 100\left[\%\right]$$

As we had to process about 120 root samples at each sampling time, we were unable to prepare thin root cross sections for all samples, which is required for an unequivocal identification of a Hartig net as another key characteristic of EM trees (Brundrett & Tedersoo, 2020). We carried out cross sections only on a randomly chosen subset of one sample per species (Figures [Link], [Link]).

For assessing the mycorrhization rates with AM fungi, the root samples that had been kept in ethanol were washed in tap water, transferred to KOH (10%), and then heated at approximately 90°C for 2 h. This resulted in tissue maceration, retaining only those parts of the roots that contained cellulose, lignin, and fungal hyphae. An additional bleaching step was added against dark tannins in the tree roots, exposing the samples to hydrogen peroxide solution (10% H_2_O_2_) containing a few drops of ammonia (25% NH_3_) for 1.5–2 h. Subsequently, the root samples were washed in tap water and acidified with drops of lactic acid for neutralization for 1 h. In a final step, the roots were stained with Trypan blue at 90°C for 3 min following the protocol by Chabaud et al. (2006). The stained fine roots were transferred in lactoglycerol and were stored in the refrigerator at 4°C.

The colonization of the root with AM was determined using a light microscope (Axiostar Plus; Zeiss, Jena, Germany). Examination of the arbuscular mycorrhization was estimated following Trouvelot et al. (1986), assessing 30 root fragments of 1 cm length each. Across all roots sampled, we calculated the frequency of arbuscular mycorrhiza (AM F %), the intensity of the arbuscular mycorrhizal colonization (AM M %), and the relative abundance of arbuscules (AM A %) as follows:

(2) $$\mathrm{AM}\;F=\frac{\text{Number of fragments with}\;\mathrm{AMF}}{\text{Total number of root fragments}\;}\times 100\left[\%\right]$$

(3) $$\mathrm{AM}\;M=\frac{\sum_{i=1}^{\text{Total number of root fragments}}\text{Proportion colonized}\;\mathrm{by}\;{\mathrm{AM}}_{i}}{\text{Total number of root fragments}}\times 100\left[\%\right]$$

(4) $$\mathrm{AM}\;A=\frac{\sum_{i=1}^{\text{Total number of root fragments}}\text{Proportion}\;{\text{with arbuscules}}_{i}\times \text{Proportion colonized}\;\mathrm{by}\;{\mathrm{AM}}_{i}}{\text{Total number of root fragments}}\times 100\left[\%\right]$$

### 2.5 Statistical analyses

All statistical analyses and plotting of figures were carried out with R, Version 4.0.3 (R Core Team, 2020). Graphs were created using the ggplot2‐package (Wickham, 2016). The impact of the sampling date, mycorrhiza host type (Myc_Type), and their interaction were analyzed with linear mixed effects models, using the ‘lmerTest’ package (Kuznetsova et al., 2017). Response variables were the different aspects of mycorrhization rates (ECT %, AM F %, AM M %, and AM A %). We tested for differences between seasons (December, March, and May) with a subsequent Tukey post‐hoc test (‘ghlt’‐function in the multcomp‐package, Hothorn et al., 2008). Tree species identity and plot were crossed random factors. To test for the effects of the plot's mixture of mycorrhiza types, levels of tree species richness per plot, and tree species (T_Species), we used the same two‐factorial models, replacing Myc_Type by Mix_Type or Spec_Rich or tree species (Species). As the morphological analysis of the roots of *Aesculus hippocastanum and Acer pseudoplatanus* did not allow to estimate ectomycorrhizal mycorrhization rates, we excluded this host species from all calculations involving EM mycorrhization rates.

For the calculation of synchrony between EM and AM frequencies within tree individuals, we calculated the Pearson correlation r between both types across the three sampling dates. Thus, the values for r had the range from −1 (EM and AM frequencies inversely related to each other, indicating maximal asynchrony) to +1 (EM and AM frequencies related to each other, indicating maximal synchrony). To assess whether r differed from zero, we calculated a linear mixed effects model including only the intercept and using tree species identity and plot as crossed random factors. We then also included the host tree's mycorrhiza type (Myc_Type, either AM or EM) and plot's mixture type (Mix_Type, mono = monotypic, i.e., AM or EM, or mix = AM+EM host tree mixture in a plot) as predictors.

At the community level, we tested synchrony separately for EM and AM frequencies, following Loreau and de Mazancourt (2008) and calculating community‐wide synchrony ϕ as follows:

$$\varphi =\frac{\sigma_{\text{Freq}}^{2}}{\;{\left(\sum_{i}^{n}\sigma_{\text{Freq Species}\;i}\right)}^{2}}$$

where $\sigma_{\text{Freq}}^{2}$ is the variance of the mycorrhization rate (either ECT or AM F) of all species in the community across all sampling dates, and σ_Freq Species *i*_ is the standard deviation of the mycorrhization rate (either ECT or AM F) of species *i* in that community of n species. In this way, the variance of community‐level mycorrhization rate is related to the synchrony of the corresponding species‐level mycorrhization rates. Synchrony ranges between 0 (complete species asynchrony), when the species‐specific mycorrhization rates are perfectly uncorrelated through time, and 1 (complete species synchrony) when the species‐specific mycorrhization rates are perfectly correlated through time. Per definition ϕ is 1 in monocultures, when plot variation is entirely the result of the mycorrhization rate of a single species (Jucker et al., 2014). We calculated ϕ for each plot and tested for differences between species diversity levels (including only two‐ and four‐species mixtures) and the interaction with the plot's mycorrhiza mixture type (Mix_Type), using tree species identity and plot nested as crossed random factors.

## 3 RESULTS

**FIGURE 1:**
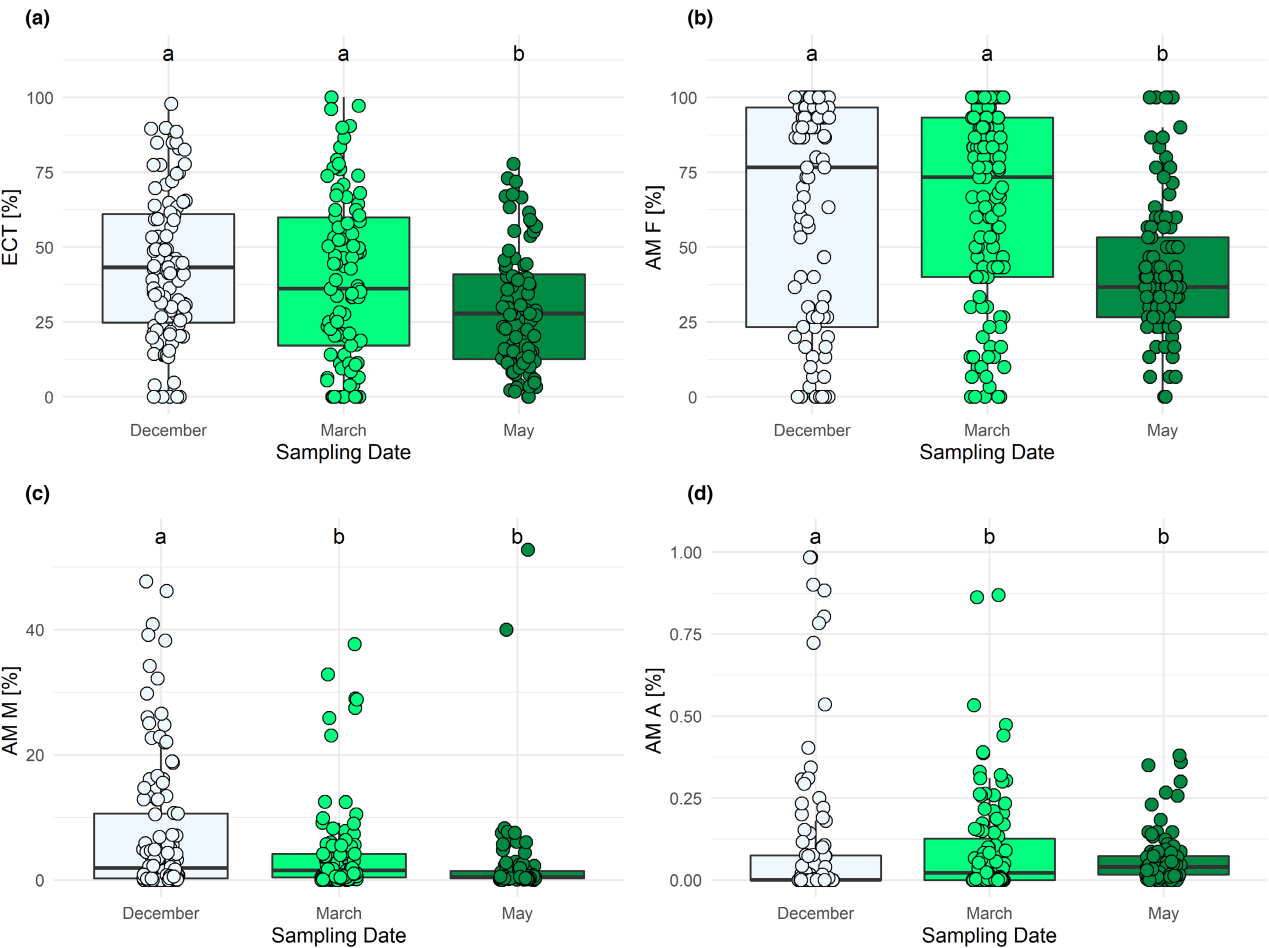
Mycorrhization rates by sampling date (December, March, and May). (a) Frequency of active ectomycorrhizal root tips (ECT), (b) frequency of arbuscular mycorrhiza (AM F), (c) intensity of the arbuscular mycorrhizal colonization (AM M), and (d) relative abundance of arbuscules (AM A). Some outliers in (d) are not shown, which are 14, 4, and 2 for December, March, and May, respectively. The results of the statistical model are shown in Table 2.

**FIGURE 2:**
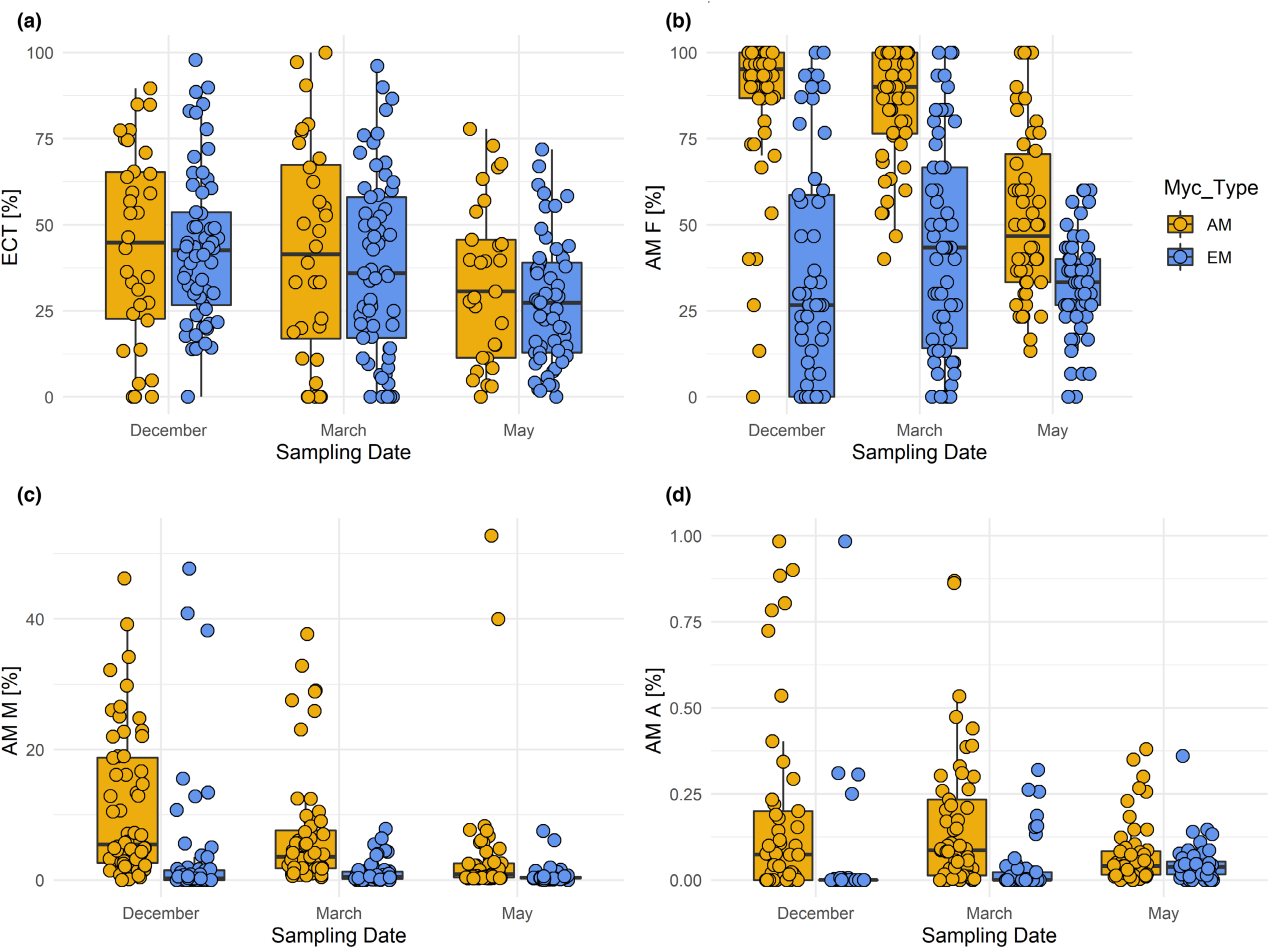
Mycorrhization rates as a function of sampling date and mycorrhiza type. (a) Frequency of active ectomycorrhizal root tips (ECT), (b) frequency of arbuscular mycorrhiza (AM F), (c) intensity of the arbuscular mycorrhizal colonization (AM M), and (d) relative abundance of arbuscules (AM A) shown by sampling date (December, March, and May) and mycorrhiza type (Myc_Type: AM = Arbuscular mycorrhiza, EM = Ectomycorrhiza). Some outliers in d) are not shown, which in December are 11 and 3 for AM and EM, in March 3 and 1, and in May 2 and 0, respectively. The results of the statistical model are shown in Table 2.

All species except for *A. hippocastanum* and *A. pseudoplatanus*, which showed only AM, the tree species were associated with both types of mycorrhiza (AM and EM) at all sampling dates (December, March, and May). The sampling date had a significant influence on mycorrhization rates both in AM and in EM (Figure 1; Table 2). The frequencies of both mycorrhiza types (ECT and AM F) decreased from December to May, with significantly lower frequencies in May than in December and March (Figure 1a,b; Table 2). AM frequency and AM colonization intensity across all sampling dates differed significantly between the host tree's preferred mycorrhiza type (Myc_Type, Figure 2; Table 2). The EM frequency (ECT) was not significantly different between AM and EM trees (Figure 2a). As expected, AM trees had consistently higher AM frequency and colonization intensity than EM trees (Figure 2b,c). While no significant interaction of sampling date and mycorrhiza type for EM frequency (ECT) was encountered (Figure 2a; Table 2), there was a significant interaction of sampling date and host mycorrhiza type for the AM frequency (Figure 2b; Table 2). Except for *S. aucuparia* (Figures S5a–d and S7a; Table S1), for all AM‐tree species, AM frequency was lowest in May, while for EM‐tree species it was lowest in December (Figure 2b; Table 2). The temporal pattern of AM colonization intensity (AM M %) followed that of AM frequency, with significant differences between sampling times and mycorrhiza types (Figure 2c; Table 2). Tree species predominately associated with EM had very low values of AM intensity at all sampling dates. The relative abundance of arbuscules (AM A %) showed no significant differences between the tree species' mycorrhiza type, but significantly differed between seasons, with decreasing values from December to May (Figure 2d; Table 2).

**TABLE 2:** ANOVA results of the linear models, relating EM frequency (ETC), AM frequency (AM F), intensity of the arbuscular mycorrhizal colonization (AM M), and relative abundance of arbuscules (AM A) to different predictors. (a) Effects of sampling date (December, March, and May). (b) Combined effects of sampling date and the host trees mycorrhiza type (Myc_Type, either AM = Arbuscular mycorrhiza or EM = Ectomycorrhiza). (c) Combined effects of sampling date and host trees species identity. For the parameter estimates of this model see Table S1. (d) Combined effects of sampling date and the plots mixture type (Mix_Type, either monotypic, i.e., AM or EM, or mixed, i.e., AM + EM host tree mixture in a plot), (e) Combined effects of sampling date and tree species richness of the plot (Spec_Rich, Mono = monoculture, Di = Two different species, Tetra = Four different species per plot). Significant *p* values are shown in bold type.

	ECT		AM F		AM M		AM A	
	*F* value	*p* value	*F* value	*p* value	*F* value	*p* value	*F* value	*p* value
(a) Sampling_Date	8.667	**.0002**	23.124	**6.13e‐10**	12.38	**7.4e‐06**	6.535	**.0017**
(b) Sampling_Date and Myc_Type								
Sampling_Date	8.580	**.0002**	25.722	**6.77e‐11**	12.944	**4.33e‐06**	6.531	**.0017**
Myc_Type	0.347	.558	160.148	**<2.2e‐16**	37.75	**3.19e‐08**	2.361	.127
Sampling_Date:Myc_Type	0.291	.747	14.235	**1.39e‐06**	2.582	.0775	0.258	.773
(c) Sampling_Date and T_Species								
Sampling_Date	9.754	**9.22e‐05**	27.896	**1.17e‐11**	14.193	**1.25e‐06**	6.718	**.0014**
T_Species	1.729	.116	25.946	**<2.2e‐16**	9.972	**1.75e‐13**	1.82	.064
Sampling_Date:T_Species	2.474	**.0031**	3.52	**3.98e‐06**	1.7	**.038**	1.243	.225
(d) Sampling_Date and Mix_Type								
Sampling_Date	8.669	**.0002**	22.967	**7.09e‐10**	12.309	**7.92e‐06**	6.504	**.0017**
Mix_Type	0.719	.404	0.011	.918	0.011	.916	0.051	.822
Sampling_Date: Mix_Type	0.962	.384	0.038	.963	0.227	.797	0.661	.517
(e) Sampling_Date and Spec_Rich								
Sampling_Date	8.609	**.0002**	23.355	**5.172e‐10**	12.298	**8.04e‐06**	6.697	**.0014**
Spec_Rich	1.5709	.227	0.136	.873	0.390	.68	1.424	.248
Sampling_Date: Spec_Rich	0.281	.890	1.463	.214	0.803	.524	2.969	**.02**

*Note*: While in (a), (b), (d), and (e) tree species identity and plot were used as crossed random factors, in (c) only plot was included as random factor.

**FIGURE 3:**
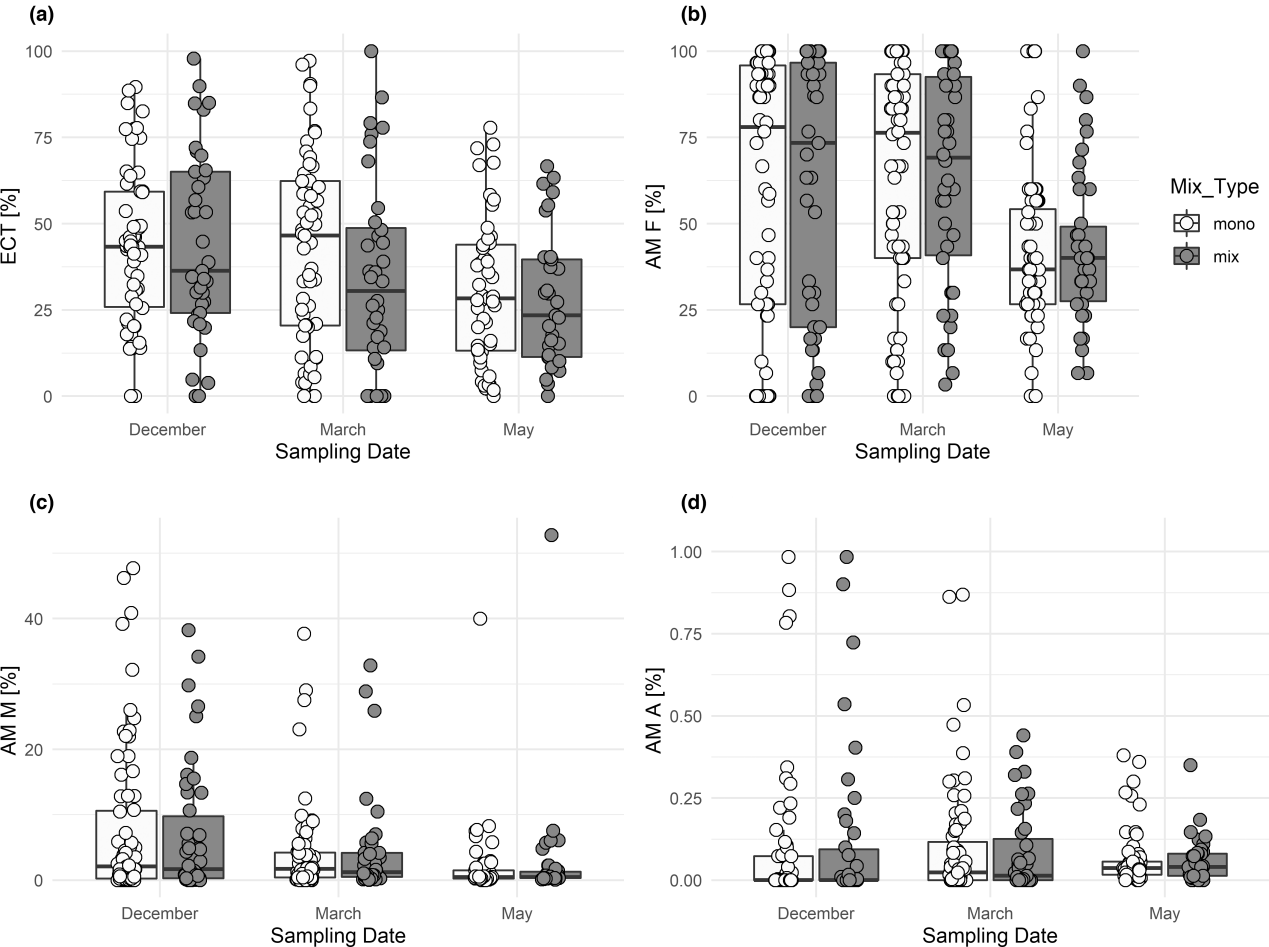
The mycorrhization rates as a function of sampling date and mixture of mycorrhiza types. (a) Frequency of active ectomycorrhizal root tips (ECT), (b) frequency of arbuscular mycorrhiza (AM F), (c) intensity of the arbuscular mycorrhizal colonization (AM M), and (d) relative abundance of arbuscules (AM A) shown by sampling date (December, March, and May) and mixture of mycorrhiza types (Mix_type), mono = monotypic, i.e., AM or EM, or mix = AM+EM host tree mixture in a plot. Some outliers in (d) are not shown, which in December are 10 and 4 for mono and mix, in March 1 and 3, and in May 1 and 1, respectively. The results of the statistical model are shown in Table 2.

The mixture of mycorrhiza types (monotypic, only AM, or only EM compared to mixed (EM + AM)) had no significant effect on any type of mycorrhization rate (Figure 3; Table 2). There was also no significant interaction with sampling date, showing that mixing tree species of different mycorrhiza types had no effect on seasonal mycorrhizal activities.

**FIGURE 4:**
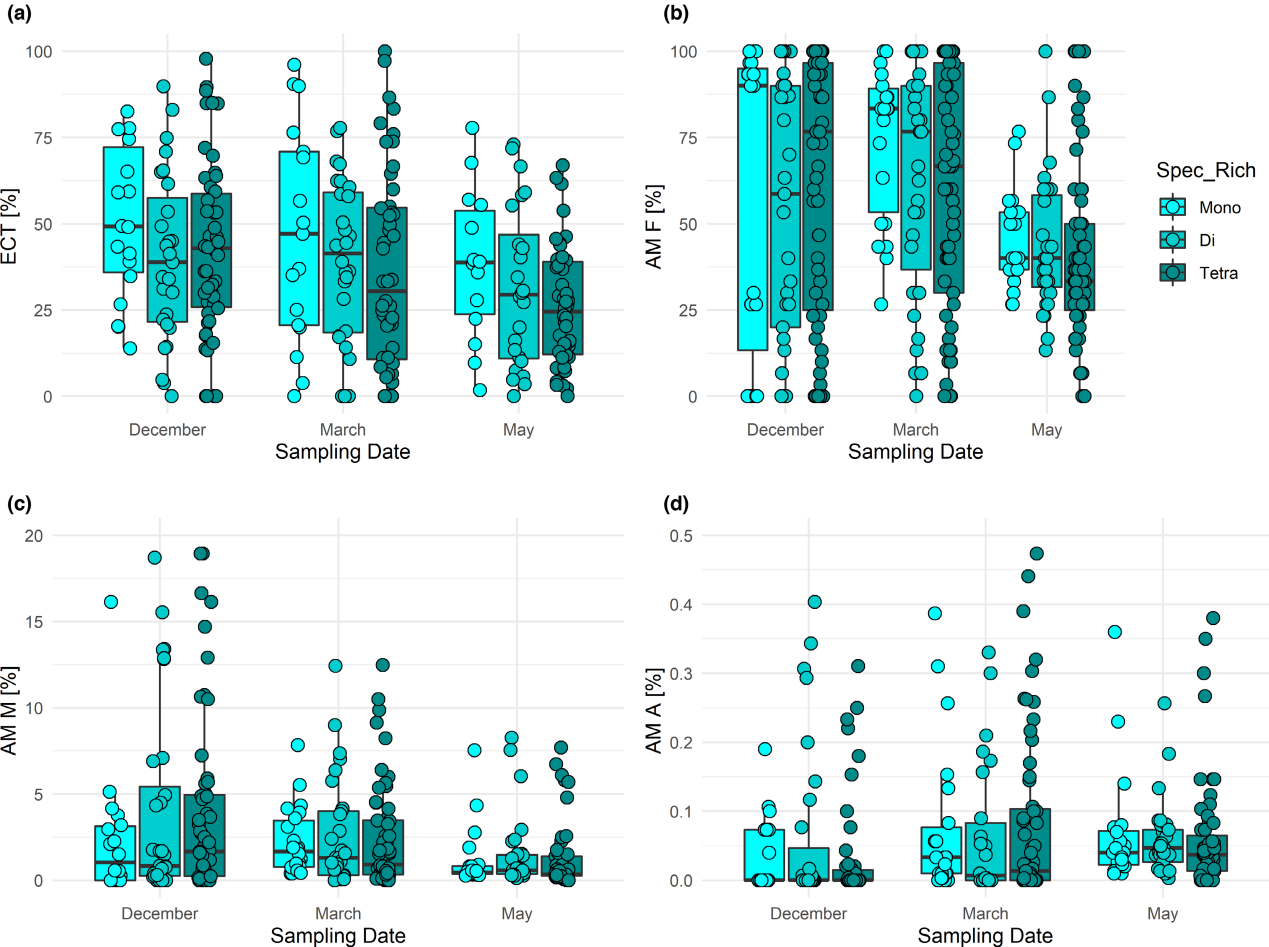
Mycorrhization rates as a function of sampling date and tree species richness. (a) Frequency of active ectomycorrhizal root tips (ECT), (b) frequency of arbuscular mycorrhiza (AM F), (c) intensity of the arbuscular mycorrhizal colonization (AM M), and (d) relative abundance of arbuscules (AM A) shown by sampling date (December, March, and May) and host species richness (Spec_Rich: Mono = tree monoculture, Di = two different tree species, Tetra = four different tree species per plot). Some outliers are not shown, which (c) in December are 5, 2, and 9 for Mono, Di, and Tetra, in March 0, 2, and 5 and in May 0, 0, and 2, respectively, and (d) in December are 5, 3 and 14 for Mono, Di, and Tetra, in March 0, 5, and 2 and in May 0, 2, and 0, respectively. The results of the statistical model are shown in Table 2.

Similarly, mostly insignificant effects on mycorrhization rates were encountered for tree species richness (Figure 4; Table 2). The three tree species richness levels (with 1, 2, or 4 species per plot) neither differed in EM frequency (ECT), nor in AM frequency, AM colonization intensity, or abundance of arbuscules. There were also no significant interactions between tree species richness and sampling date, except for the abundance of arbuscules (Figure 4d; Table 2). This interaction was brought about by a higher abundance of arbuscules in monocultures than in two‐species mixtures in March and opposing pattern in May (Figure 4d; Table 2).

**FIGURE 5:**
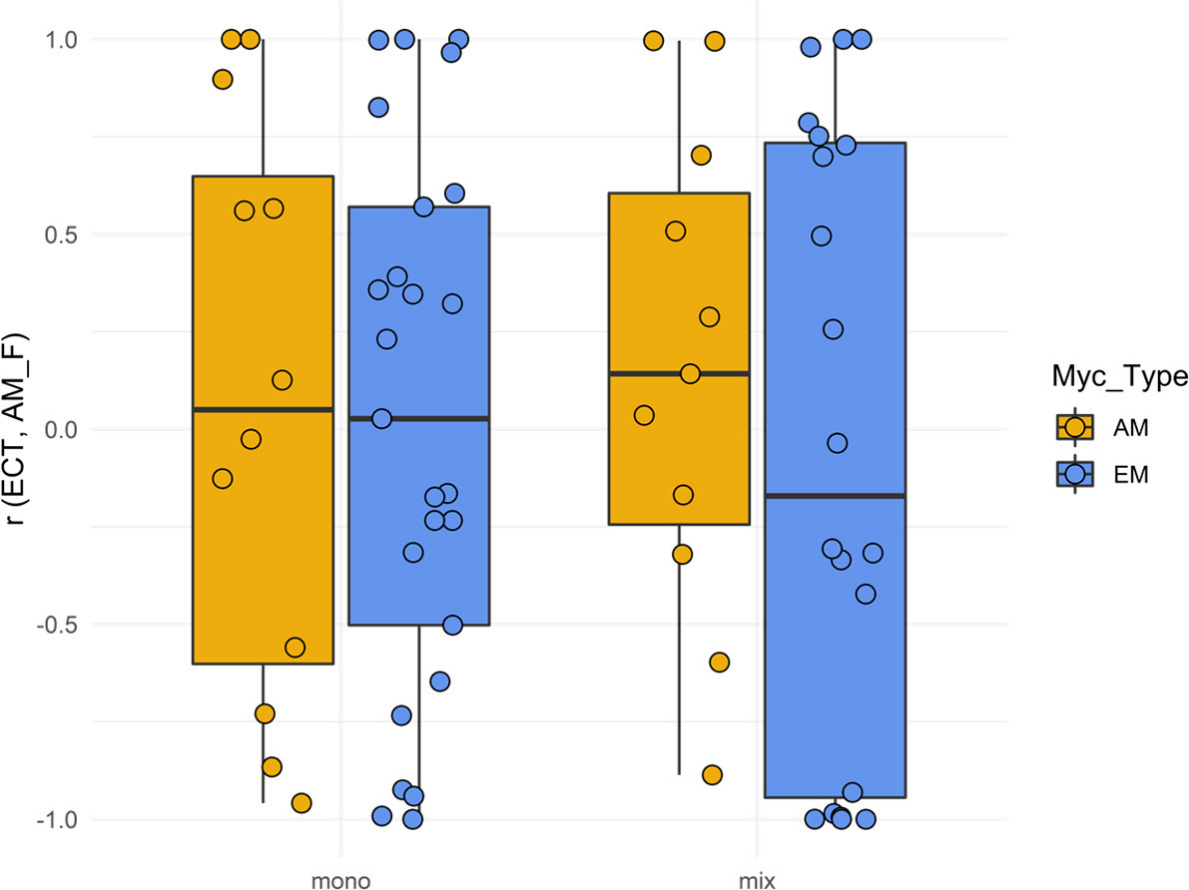
Correlation between mycorrhization by EM (ECT) and AM (AM_F) within tree individuals as assessed by Pearson correlation coefficient *r*. *r* was not significantly different from zero, neither overall, nor by the host tree's mycorrhiza type (Myc_Type, either AM or EM) or by the host tree mixture (Mix_Type), mono = monotypic, i.e., AM or EM, or mix = AM+EM host tree mixture in a plot. The results of the statistical model are shown in Table 3.

Pearson correlation between mycorrhization by EM (ECT) and AM (AM F) within tree individuals was not significantly different from zero (Figure 5). Thus, the frequency of AM and EM observed in a single tree varied independently of each other. There was also no difference from zero when AM host trees were compared with EM trees or monotypic plots with mixed mycorrhiza type (Figure 5).

**FIGURE 6:**
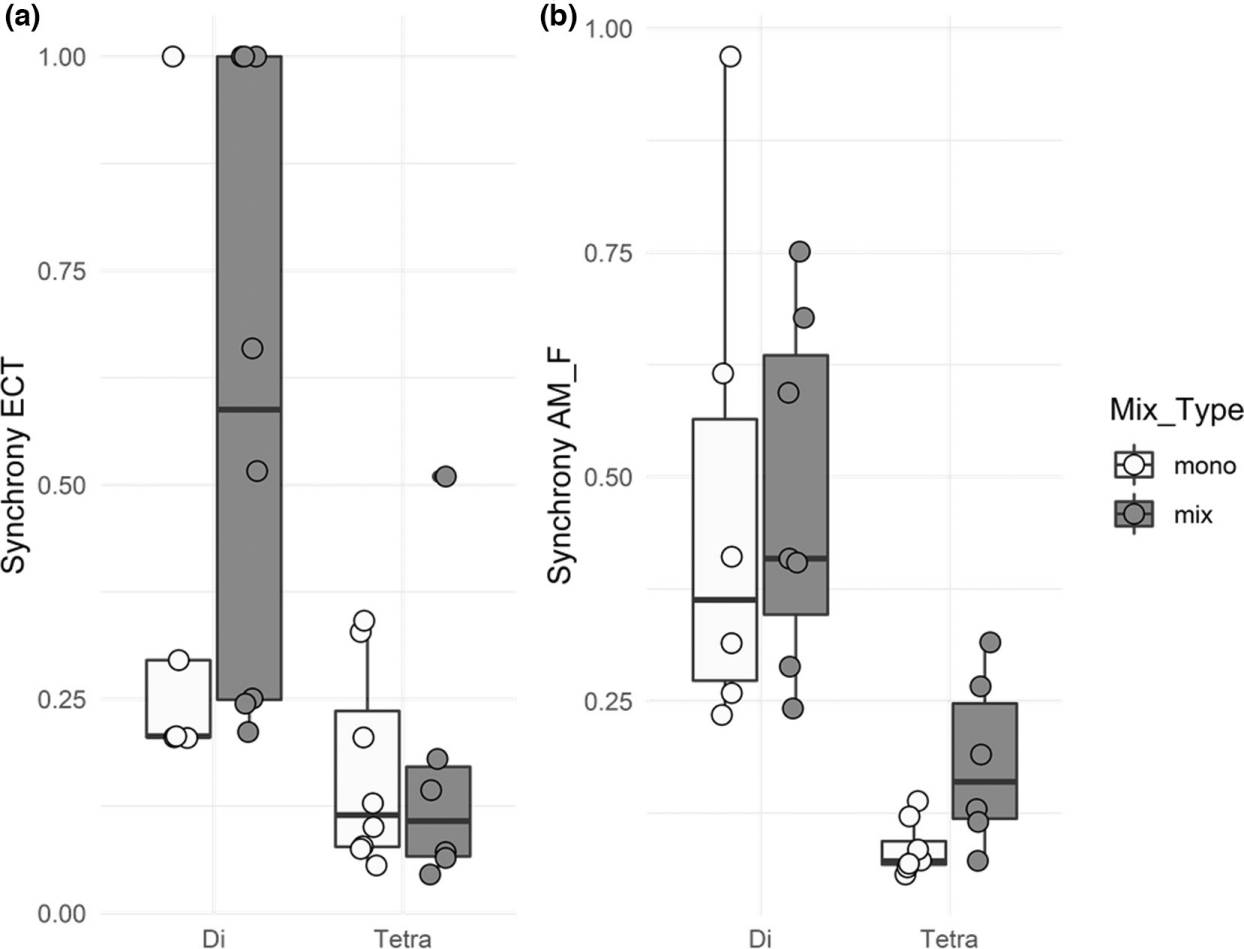
Community‐wide synchrony ϕ of mycorrhization rates for the frequency of (a) EM (ECT) and (b) AM (AM_F) as a function of plot tree species richness (Di and Tetra for two and four tree species per plot, respectively) and the mixture of mycorrhiza types (monotypic, i.e., AM or EM, or mixed, i.e., AM+EM). The values of synchrony range between 0 (perfect asynchrony) and 1 (perfect synchrony). Both the ECT and the AM_F synchrony were significantly higher in plots with two tree species than in plots with four tree species. The results of the statistical model are shown in Table 3.

**FIGURE 7:**
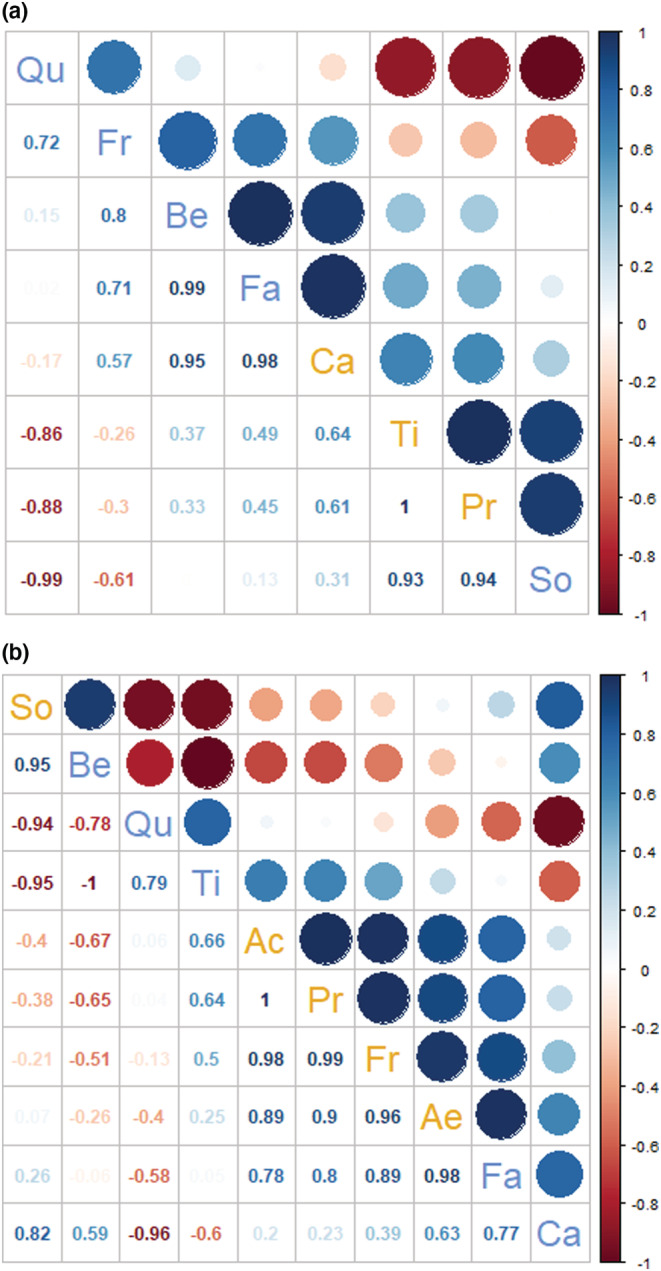
Correlation matrix of frequency of (a) active ectomycorrhizal root tips (ECT) and (b) arbuscular mycorrhiza (AM F) of the tree species. Pearson correlations range from −1 to 1, indicating perfect negative and positive correlations, respectively, across seasons between each pair of species. Ac = *Acer pseudoplatanus*, Ae = *Aesculus hippocastanum*, Fr = *Fraxinus excelsior*, Pr = *Prunus avium*, So = *Sorbus aucuparia* as trees predominantly associated with arbuscular mycorrhiza (AM, in orange fonts) and Be = *Betula pendula*, Ca = *Carpinus betulus*, Fa = *Fagus sylvatica*, Qu = *Quercus petraea*, and Ti = *Tilia platyphyllos* as tree species predominantly associated with ectomycorrhiza (EM, in blue fonts).

While the frequencies of EM and AM were not synchronized within trees, each of these two types of mycorrhization rates showed some degree of synchrony across all species at the community level, that is in a given plot (Figure 6). While synchrony was not dependent on whether the species in a community were solely composed of a single mycorrhiza type (AM or EM) or of a mix of them (AM+EM) in a plot, synchrony decreased with increasing species richness (Figure 6; Table 3). The underlying reason for this decrease in synchrony when more species occurred together was that many pairwise correlations in EM frequency (ECT, Figure 7a) or AM frequency (AM F, Figure 7b) were only weak or negative. This was also the case within the group of tree species of the same mycorrhizal type (EM or AM trees). For example, within EM tree species, the EM frequencies of *B. pendula* and *F. sylvatica* were highly synchronous (*r* = +0.99), while those of *Q. petraea* and *T. platyphyllos* were highly asynchronous (*r* = −0.86; Figure 7a). Similarly, asynchronous was the EM frequencies of *Q. petraea* with the AM species *S. aucuparia* (*r* = −0.99). The same patterns were encountered for AM frequencies (Figure 7b). While the AM frequencies within the AM species *A. hippocastanum*, *F. excelsior*, *P. avium*, and *A. pseudoplatanus* were highly synchronous (*r* between +0.89 and +0.99), their AM frequencies were negatively correlated or uncorrelated with those of *S. aucuparia* (*r* between −0.40 and +0.06). Similarly, the AM frequencies of the EM species *C. betulus* and *Q. petraea* were strong asynchronous (*r* = −0.96), while those of *Q. petraea* and *T. platyphyllos* were relatively synchronous (*r* = +0.79).

**TABLE 3:** Synchrony φ in mycorrhization by EM (ECT) and AM (AM_F) as a function of the plot tree species diversity level (Spec_Rich, Mono = monoculture, Di = Two different species, Tetra = Four different species per plot) and mixture of mycorrhiza types (Mix_Type, either AM = Arbuscular mycorrhiza or EM = Ectomycorrhiza). Significant *p* values are shown in bold type.

	Synchrony in ECT		Synchrony in AM_F	
	*F* value	*p* value	*F* value	*p* value
Spec_Rich	12.177	**.002**	27.775	**2.393e‐05**
Mix_Type	1.1287	.299	0.721	.405
Spec_Rich:Mix_Type	1.146	.295	0.391	.538

*Note*: φ was calculated according to Loreau and de Mazancourt (2008).

## 4 DISCUSSION

Our study on young tree species with dual mycorrhization revealed a clear temporal variation both for the frequency of ectomycorrhizal and arbuscular mycorrhizal colonization rates, with a general decline from winter (December) to early summer (May). Sampling date had an important effect on all response variables, thus confirming our first hypothesis that mycorrhization rates of both AM and EM depend on time of sampling. In contrast, we only found limited support for our second hypothesis that seasonal patterns in mycorrhization rates differed between AM and EM tree species. These results seem to be tightly associated with, on the one hand, the species phenology which followed the general seasonality of the temperate zone and supposedly ensued a seasonal variation of belowground carbon allocation to the tree roots (Epron et al., 2011, 2012). On the other hand, the weather conditions within seasons, such as the extraordinarily warm and dry spell in May, also affected mycorrhization rates. We do not know whether these morphologically assessed mycorrhization rates are reflected in changes in mycorrhizal fungal community composition in this period, because molecular analyses of root samples were done only for samples taken in December (Heklau et al., 2021). While Heklau et al. (2021) described a significant relationship between AM colonization in the root system and OTU richness of Glomeromycota across different tree species, other studies attributed the variation in AM composition more to space than host identity or season. Analyzing the AM community composition of two understory forest plants (the deciduous shrub *Euonymus europaea* and the evergreen climber *Hedera helix*) over different seasons, Grünfeld et al. (2021) found that the physical distance between samples exerted a stronger influence on AM community composition than either sampling time or host specificity. In consequence, they encountered only a low seasonality in AM community composition. However, Grünfeld et al. (2021) based these conclusions on molecular analyses without testing morphologically if arbuscules and vesicles as structures of active AM were present in the cortex cells of the host organisms.

While the seasonal patterns did not differ between the two groups of AM and EM tree species, some opposing trends were observed within each of these groups. While within EM tree species (particularly *B. pendula*, *C. betula*, and *F. sylvatica*) EM frequency declined from December to May, their AM frequency increased from December to March, while *Q. petraea* had relatively low AM frequency at all sampling times. Thus, among all tested EM species, *Q. petraea* comes closest to what would be expected to be a typical EM species. Additionally, arbuscules were never encountered in the roots of *Q. petraea*. Generally, the EM frequencies of typical EM trees were not different from AM trees, which might be explained by the experimental site of the MyDiv experiment. As the trees were planted on former arable land, lower EM mycorrhization rates might be explained by the absence of sufficient spores of their preferred EM symbiosis fungal partner species. This view is supported by the absence or rarity of typical fungal ectomycorrhizal specialists in forests at this early stage of the MyDiv experiment (Heklau et al., 2021). In our study, there was a common trend across all AM and all EM tree species (hypothesis 2), species within these mycorrhiza types responded quite idiosyncratically. An example of this was *S. aucuparia*. Young trees of S*. aucuparia* (AM species) had a relatively high AM frequency (ca. 90%) throughout all seasons, but relatively low AM colonization intensities (around 4.6%). Raspé et al. (2000) reported AM infection levels of *S. aucuparia* to vary with habitat and tree age. While trees on mining spoil in northern Bohemia showed mycorrhization rates of 13%–40%, higher rates were observed in Germany (30%–60%) and lower ones in North America (10%–20%) in nursery plants (Morrison et al., 1993). Vosátka (1989) studied adult trees of *S. aucuparia* and *A. pseudoplatanus* during two vegetation seasons in a mountain region affected by SO_2_ emissions in northern Bohemia. Trees with grass undergrowth had a significantly higher mycorrhizal infection (*Sorbus* 34%–40%, *Acer* 15%–31%) than trees without grass undergrowth (*Sorbus* 13%–25%, *Acer* 3%–16%). It seems that both species benefited from the mycorrhizal‐spillover effect of AM because glomeromycotan fungi are mostly host unspecific (McGonigle & Fitter, 1990).

We had expected that the dual mycorrhization of tree species would allow trees to switch their mycorrhiza type (from EM to AM or vice versa) according to the environmental conditions. This would have resulted in a negative correlation of both mycorrhiza types within single trees, for which we found no support. Thus, we have to reject our third hypothesis, as correlations between the frequencies of EM (ECT) and AM (AM F) at the tree level were not different from zero. We found a general decrease of tree‐community level synchrony both for the frequencies of AM and EM with increasing species richness. In contrast, synchrony of none of the two types was dependent on whether the trees were planted in monotypic stands of one mycorrhiza type or in mixtures of both types. Thus, we can only partially confirm the fourth hypothesis. Overall, our findings indicate that asynchrony was neither brought about by individual trees switching their preferred mycorrhiza type (hypothesis 3) nor by the mycorrhiza type of the host tree species, as neither AM nor EM trees intensified AM or EM mycorrhization at different times in the season compared to EM and AM trees, respectively (hypothesis 4). Instead, asynchrony was caused by contrasting mycorrhization of different species, irrespective of their preferred mycorrhiza type.

## 5 CONCLUSION

So far, tree‐community asynchrony has been mainly evoked as potential mechanism to explain the effects of diversity on ecosystem functioning, such as productivity and stability of productivity (Craven et al., 2018; Isbell et al., 2015; Jucker et al., 2014; Schnabel et al., 2019). We could now demonstrate that asynchrony in young tree communities also applies to biotic interactions, using the example of mycorrhiza. We provided evidence that the frequency of mycorrhization changes with sampling time. While we consider it highly probable that macroclimate is the underlying driver of this seasonally induced variation, we have no evidence of whether these effects are caused directly by temperatures or by belowground carbon transfer from the tree roots. More importantly, the changes in mycorrhization rates were stronger when combining different host species rather than different types of hosts (that is EM or AM host trees). As a consequence, increasing species richness at the community scale increases asynchrony in mycorrhization rates. Thus, the more host species are part of the community, the higher the interspecific competition and the higher the constant efforts for a mycorrhization as high as possible in any of the host species. An important aspect of our findings was that not only potential AM hosts contributed to asynchrony in AM mycorrhization rates, but also EM hosts. Vice versa, also AM host trees played a role in EM frequency, underlining the important finding of dual mycorrhization in most of our host trees (Heklau et al., 2021). While we have shown this for young trees on nutrient‐rich arable soil, further analyses should follow on nutrient‐poor forest soils and with other potential host species that might cause spill‐over such as perennial herbs or grasses. Moreover, what we do not know yet is whether the same fungal partner taxa form mycorrhizal associations with different trees at different times of the season. Answering this question would require repeated sequencing of the host trees' roots across seasons. If this assumption was true, that is that the same fungal taxa use the resources offered by different tree species at different times of the year, host tree diversity would provide overall stability in resource supply of the fungal partner. In this case, by increasing community asynchrony in mycorrhization tree diversity would contribute a completely new dimension to forest ecosystem functioning.

## AUTHOR CONTRIBUTIONS

**Heike Heklau:** Conceptualization (equal); data curation (equal); formal analysis (supporting); methodology (equal); supervision (lead); validation (supporting); visualization (supporting); writing – original draft (supporting); writing – review and editing (equal). **Nicole Schindler:** Formal analysis (equal); investigation (supporting); methodology (supporting); writing – review and editing (equal). **Nico Eisenhauer:** Project administration (equal); resources (equal); writing – review and editing (equal). **Olga Ferlian:** Project administration (equal); writing – review and editing (equal). **Helge Bruelheide:** Conceptualization (equal); formal analysis (equal); funding acquisition (lead); project administration (equal); resources (equal); supervision (equal); visualization (lead); writing – original draft (lead); writing – review and editing (equal).

## FUNDING INFORMATION

This study was supported by the German Centre for Integrative Biodiversity Research (iDiv) Halle‐Jena‐Leipzig, funded by the German Research Foundation (FZT 118).

## CONFLICT OF INTEREST STATEMENT

The authors have no conflict of interest to declare.

## Supporting information


Figure S1.

Figure S2.

Figure S3.

Figure S4.

Figure S5a.

Figure S5b.

Figure S5c.

Figure S5d.

Figure S6.

Figure S7.

Figure S8.

Figure S9.

FigureCaptions.

Table S1.

## Data Availability

Data for mycorrhization rates, for both ectomycorrhizal and arbuscular mycorrhiza, are available here: https://doi.org/10.5061/dryad.dfn2z354f.
