# Supplementary figures and images for: Temporal variation of mycorrhization rates in a tree diversity experiment
Source: Ecol Evol. 2023 Apr 19;13(4):e10002. doi: 10.1002/ece3.10002 (PMC10115898; doi:10.1002/ece3.10002)

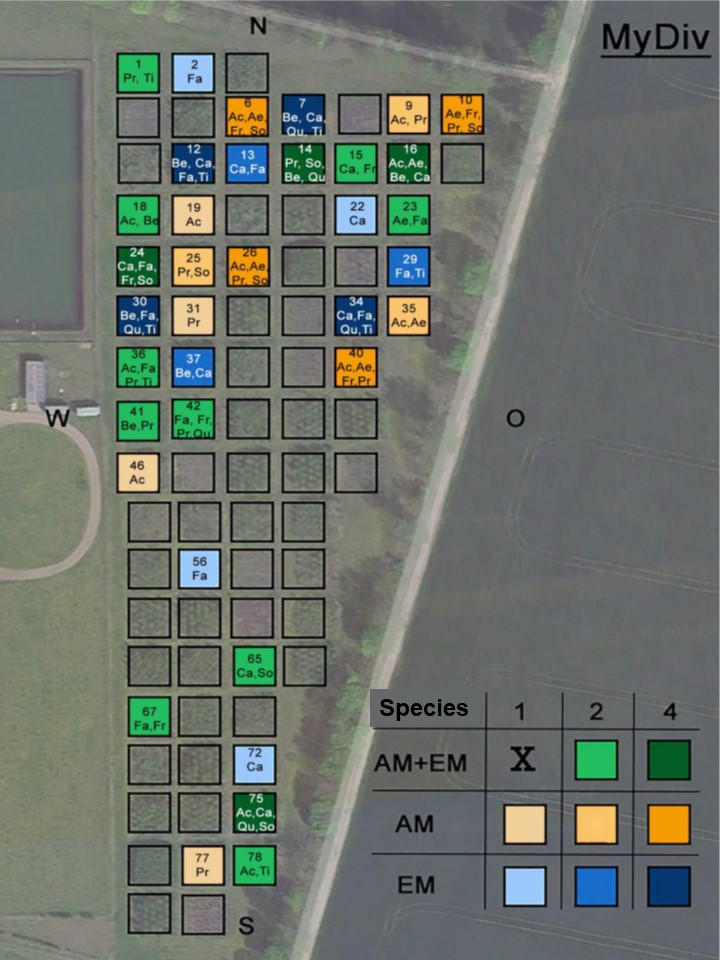

Supplement: Supplementary file 1 — Figure S1. [file ECE3-13-e10002-s009.jpg]

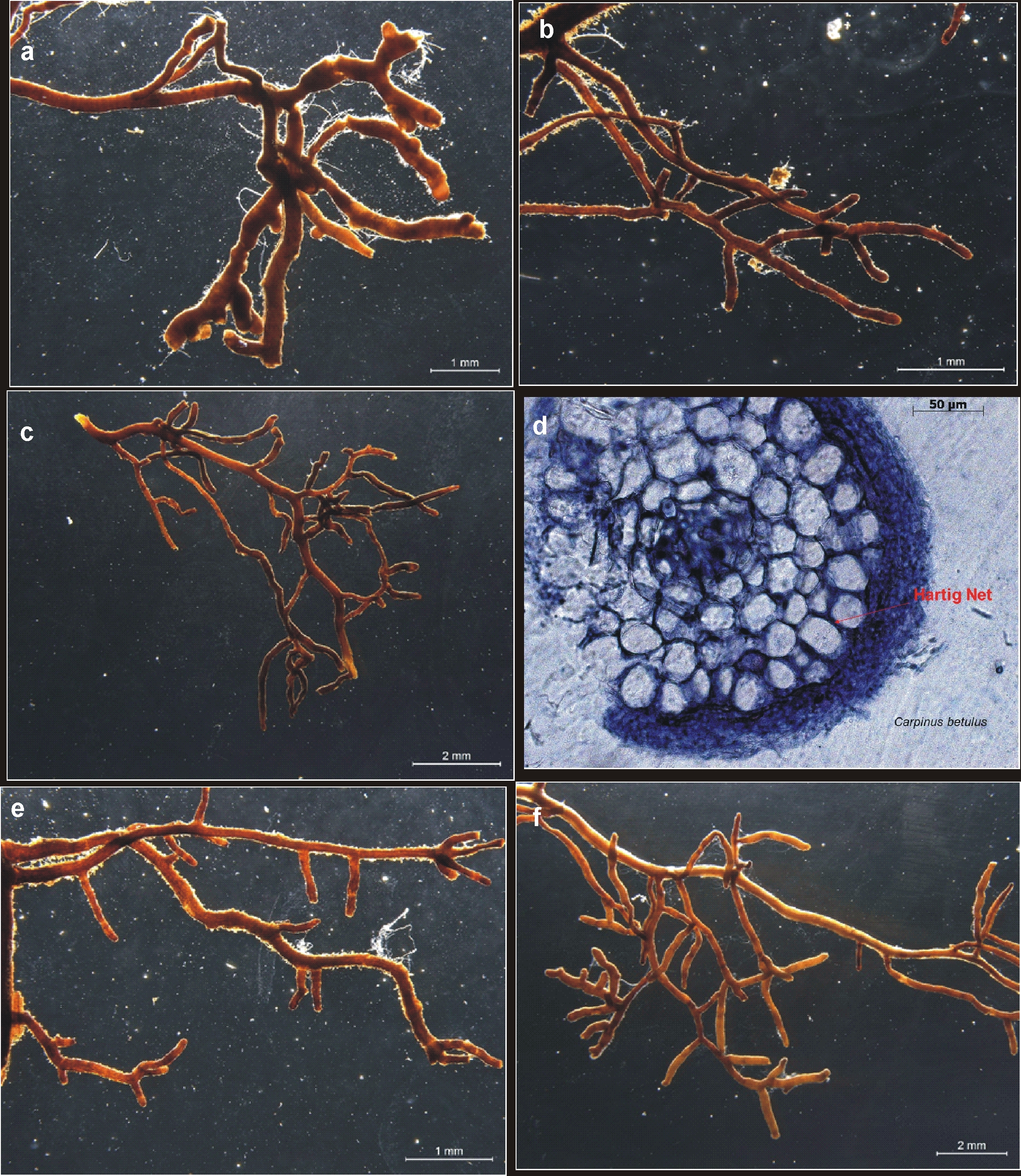

Supplement: Supplementary file 2 — Figure S2. [file ECE3-13-e10002-s002.jpg]

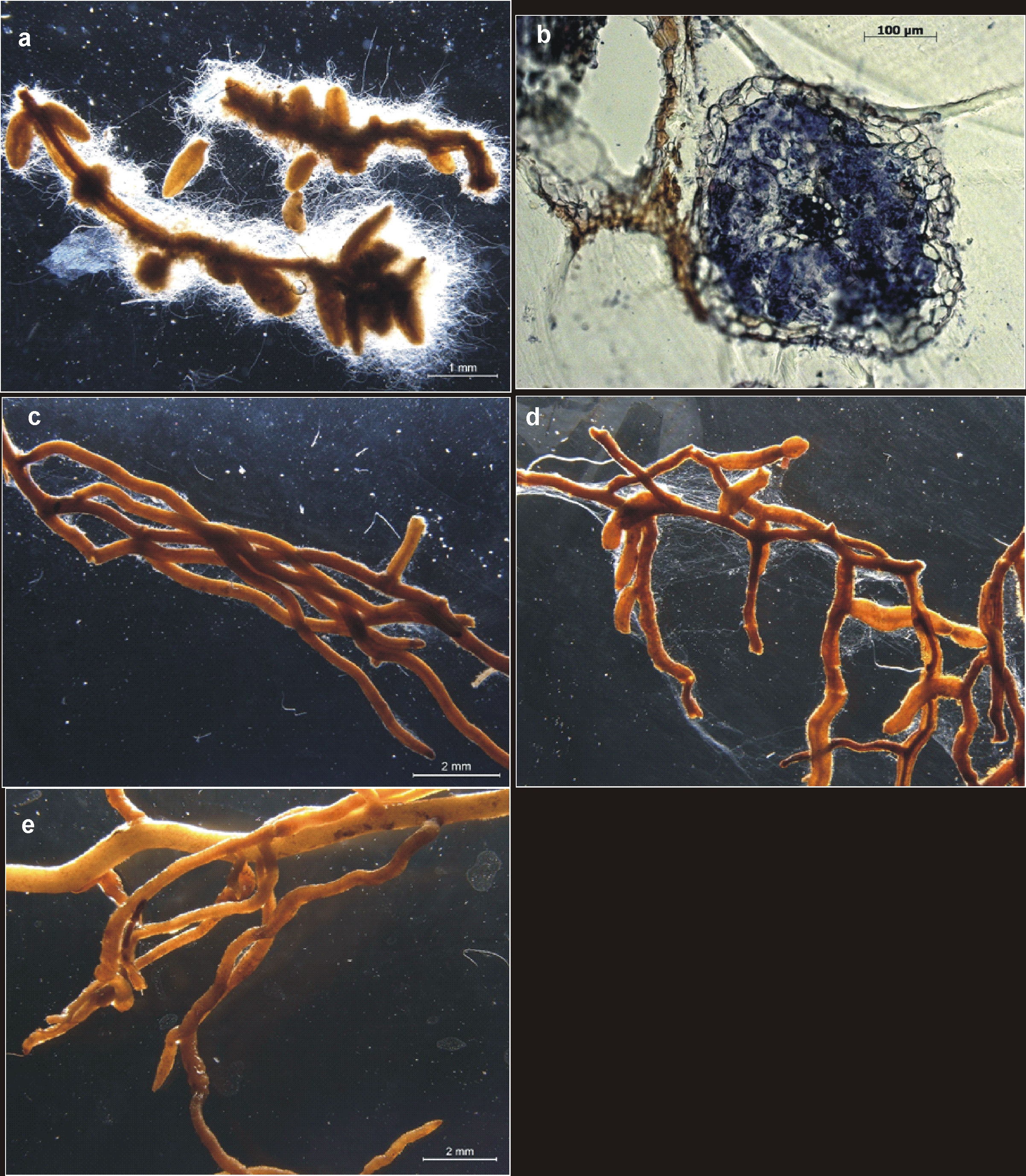

Supplement: Supplementary file 3 — Figure S3. [file ECE3-13-e10002-s008.jpg]

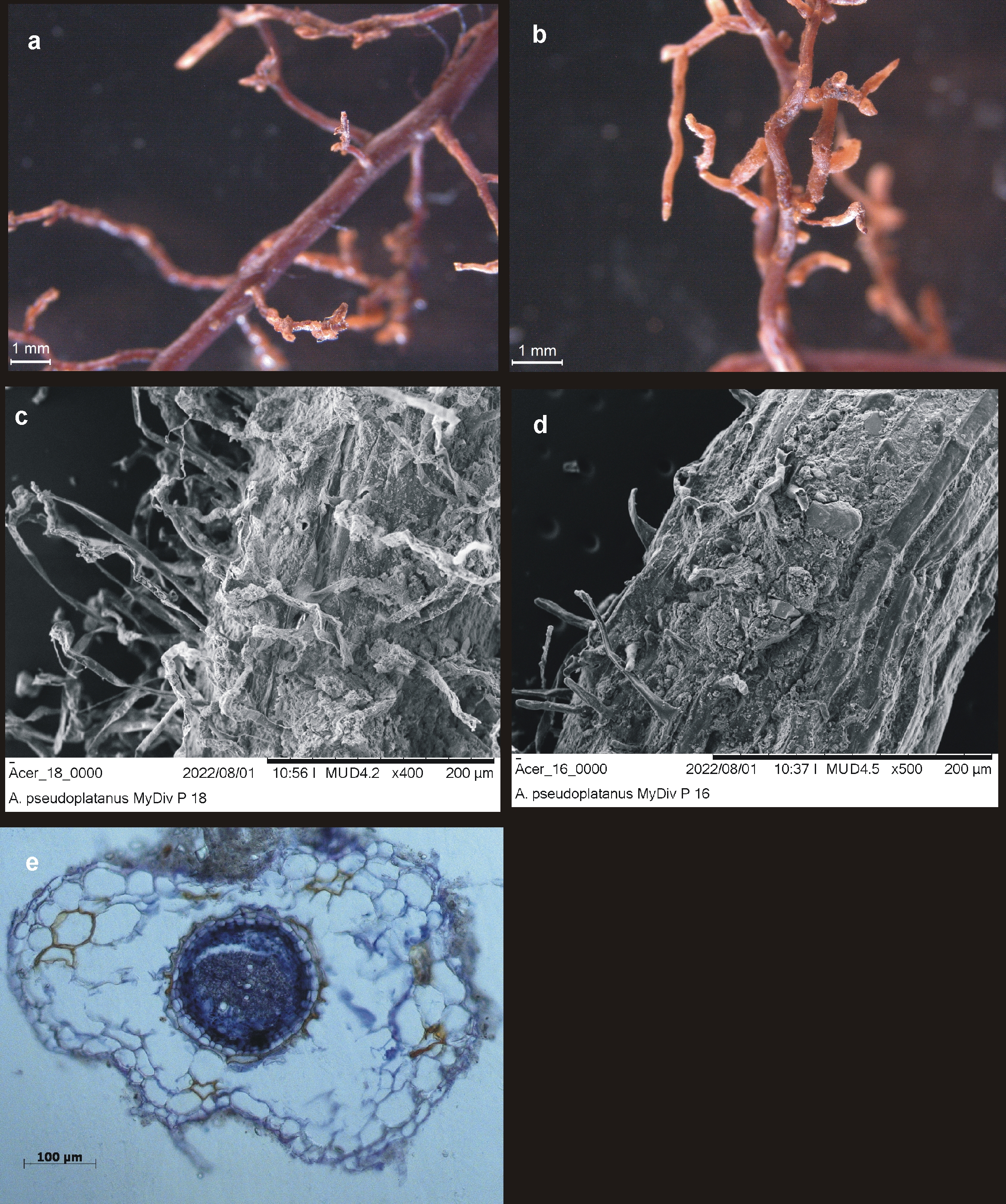

Supplement: Supplementary file 4 — Figure S4. [file ECE3-13-e10002-s007.jpg]

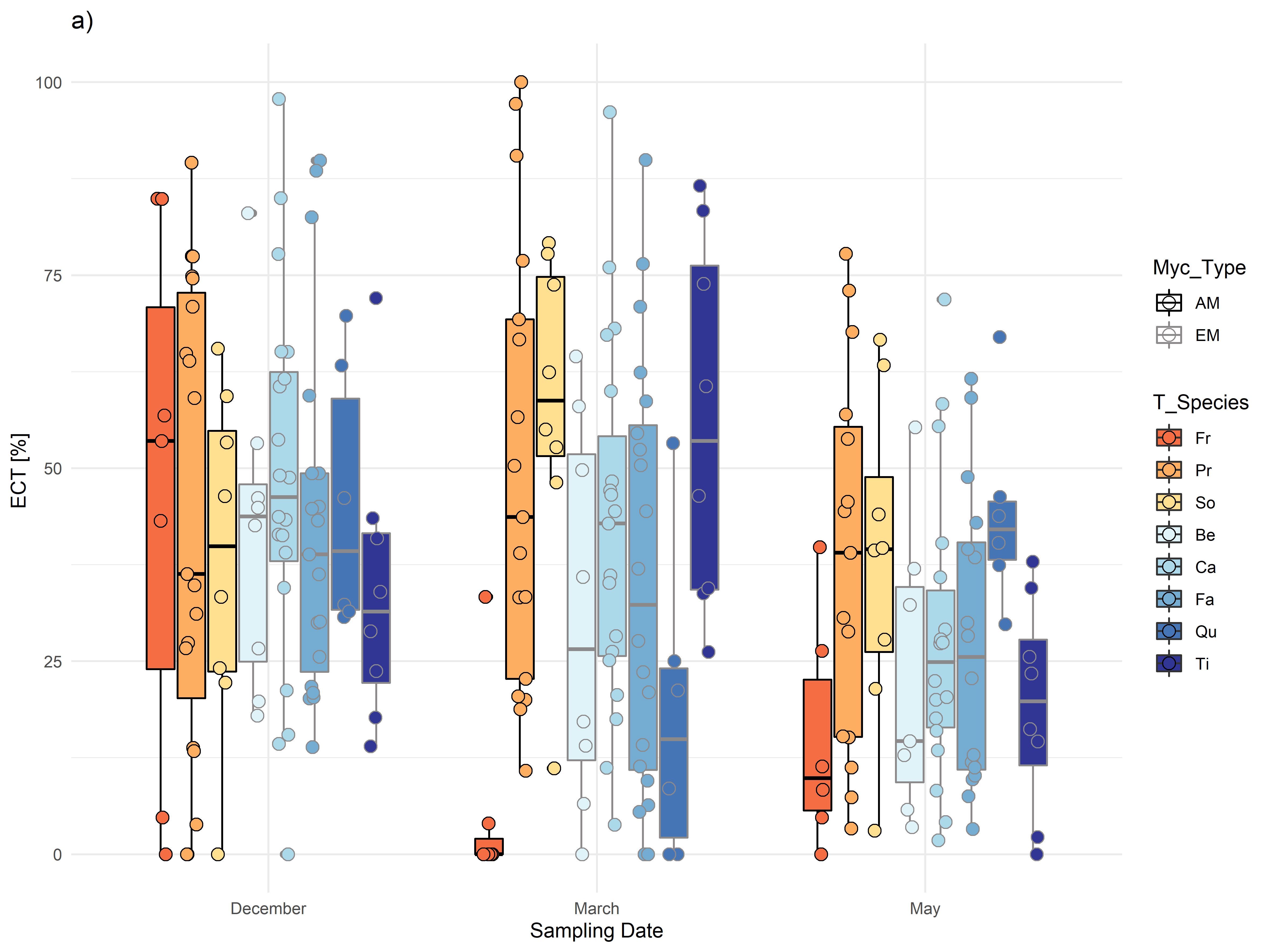

Supplement: Supplementary file 5 — Figure S5a. [file ECE3-13-e10002-s011.png]

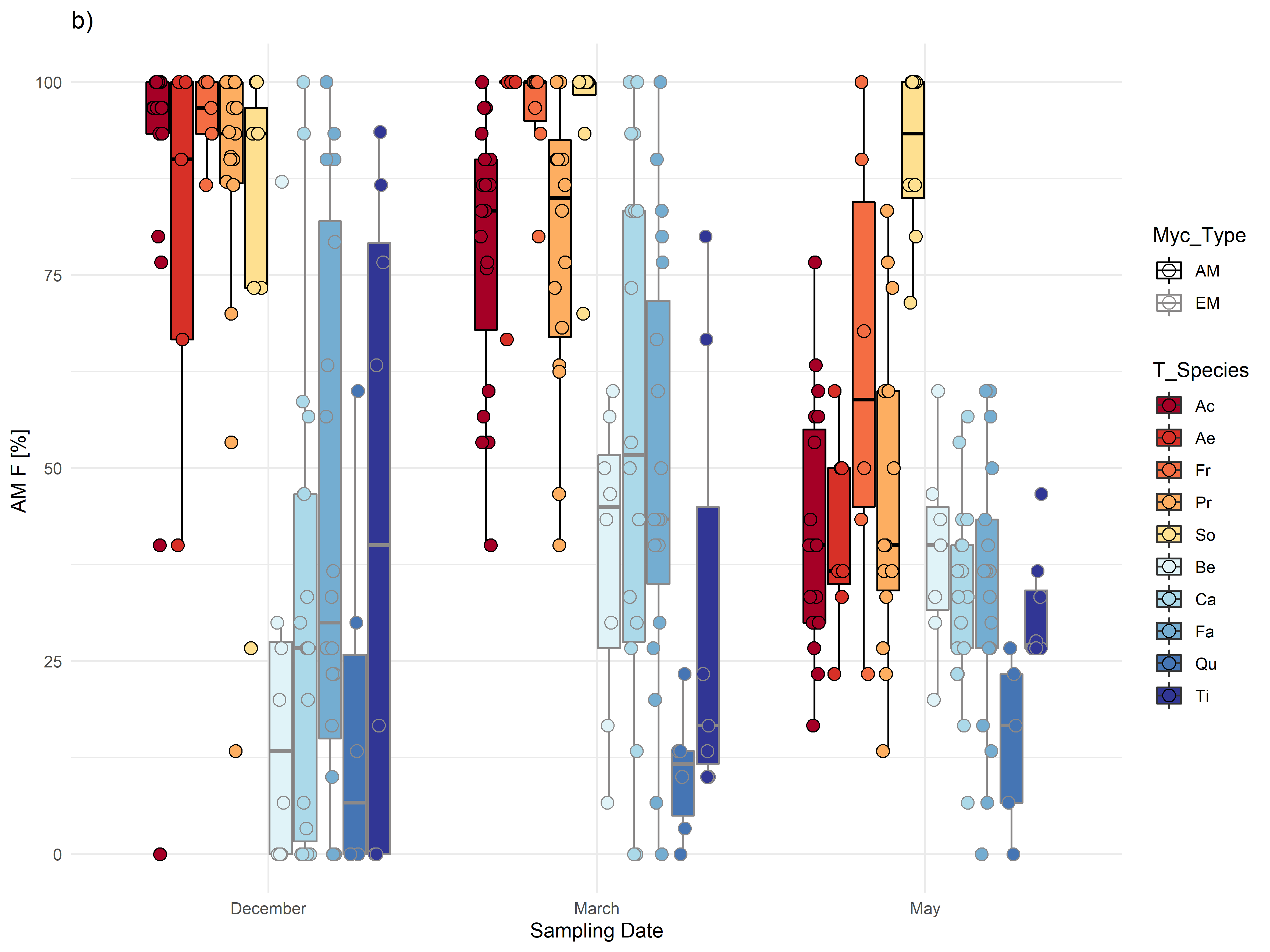

Supplement: Supplementary file 6 — Figure S5b. [file ECE3-13-e10002-s005.png]

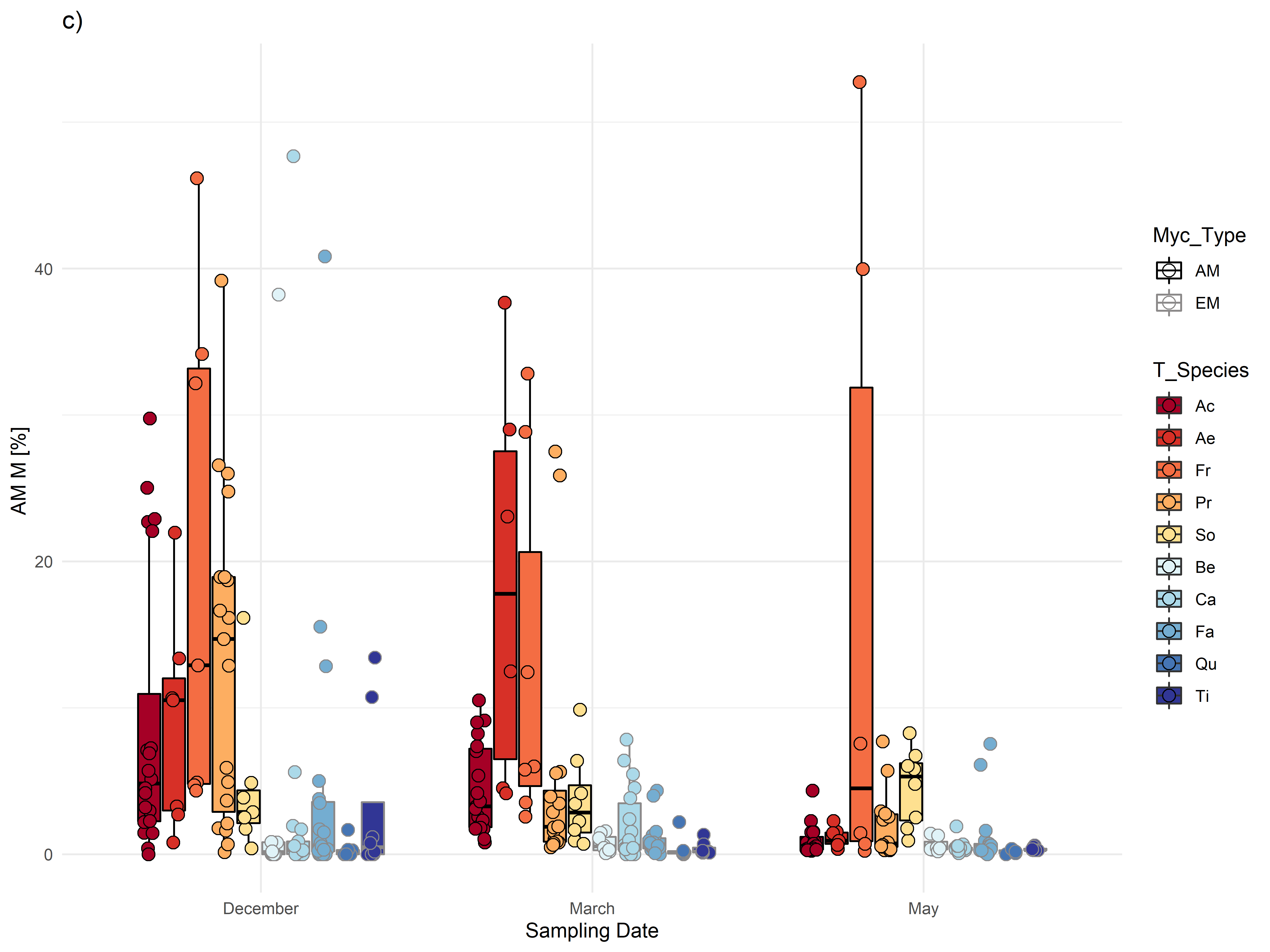

Supplement: Supplementary file 7 — Figure S5c. [file ECE3-13-e10002-s013.png]

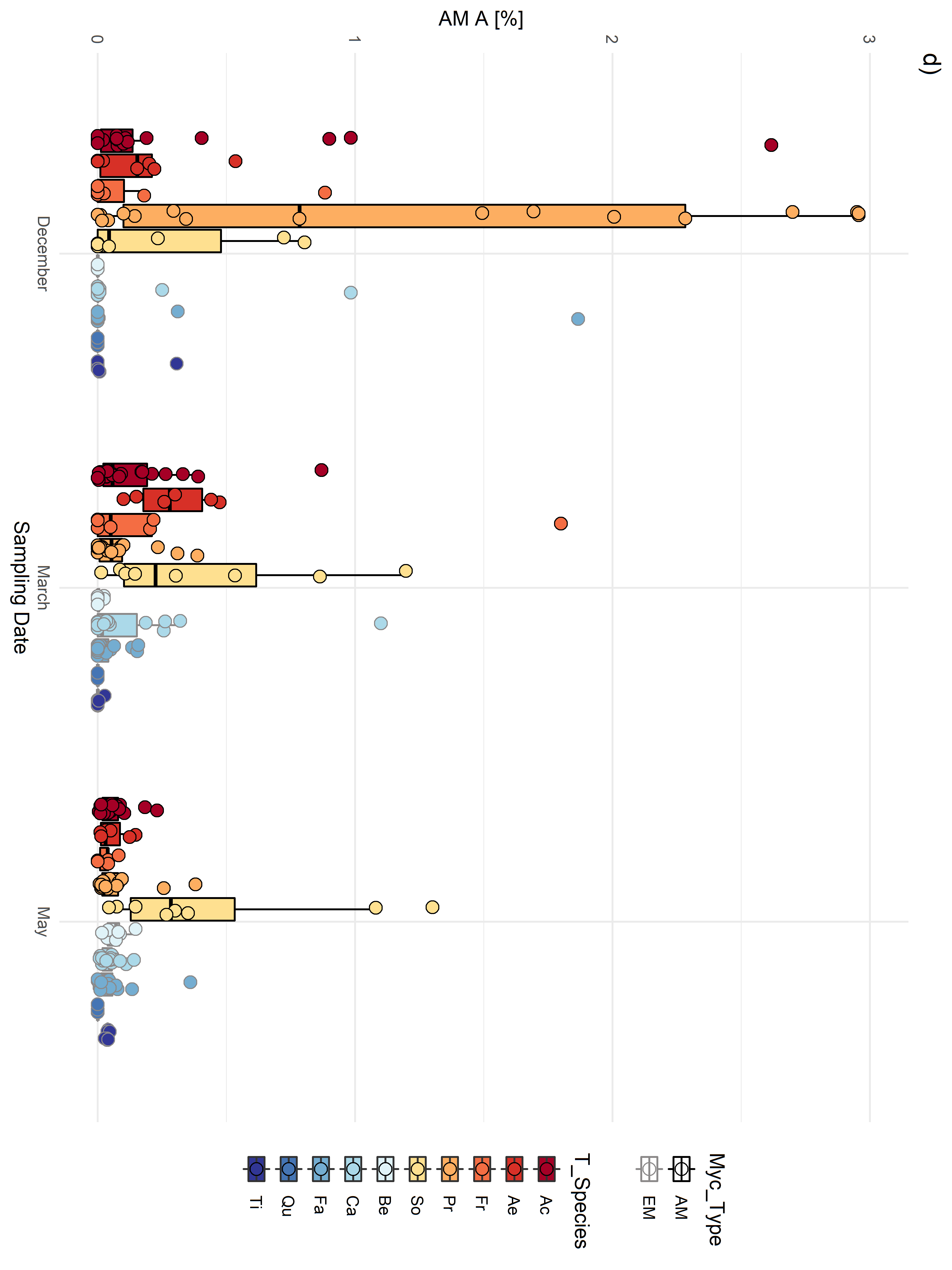

Supplement: Supplementary file 8 — Figure S5d. [file ECE3-13-e10002-s003.png]

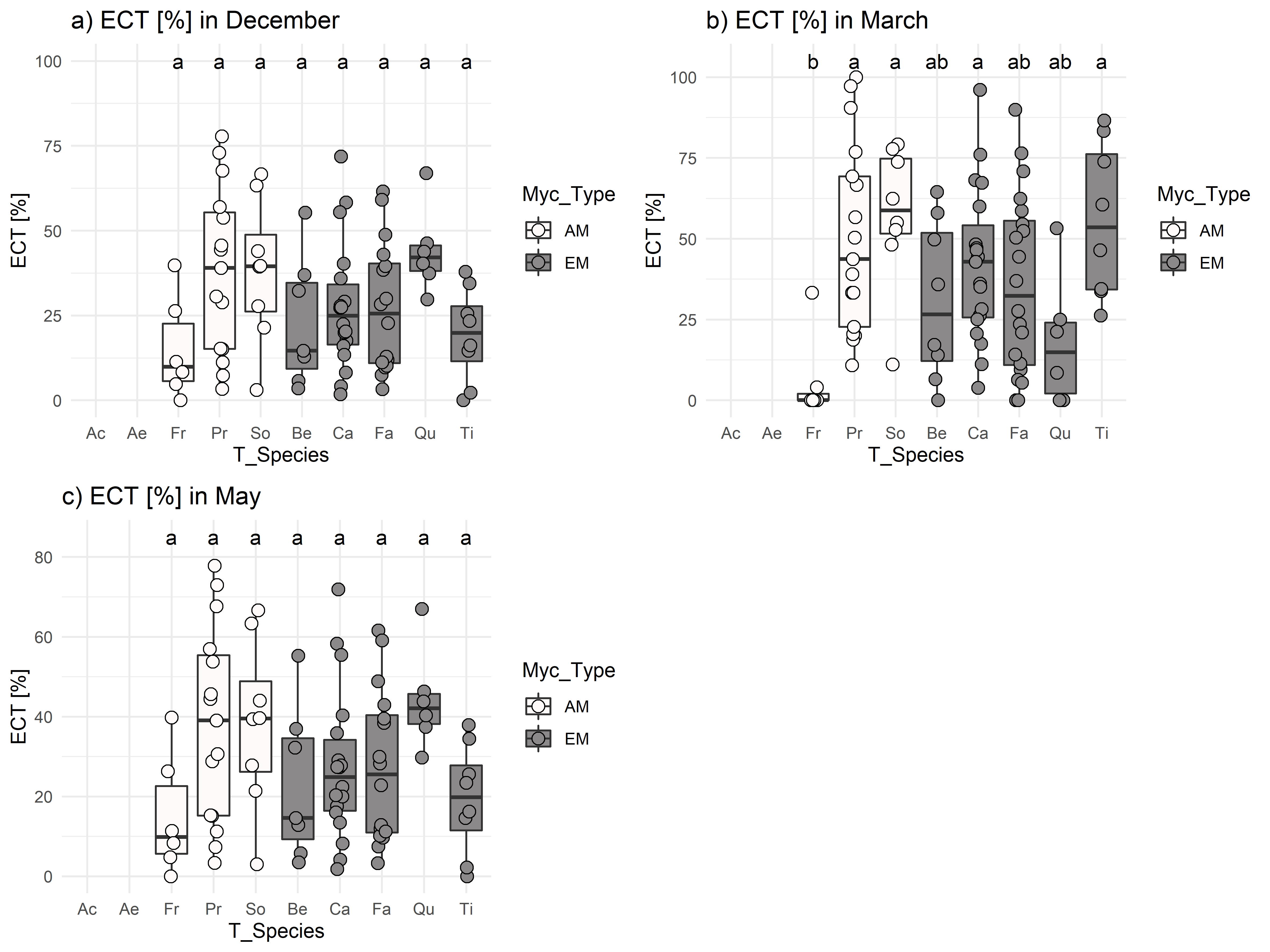

Supplement: Supplementary file 9 — Figure S6. [file ECE3-13-e10002-s001.png]

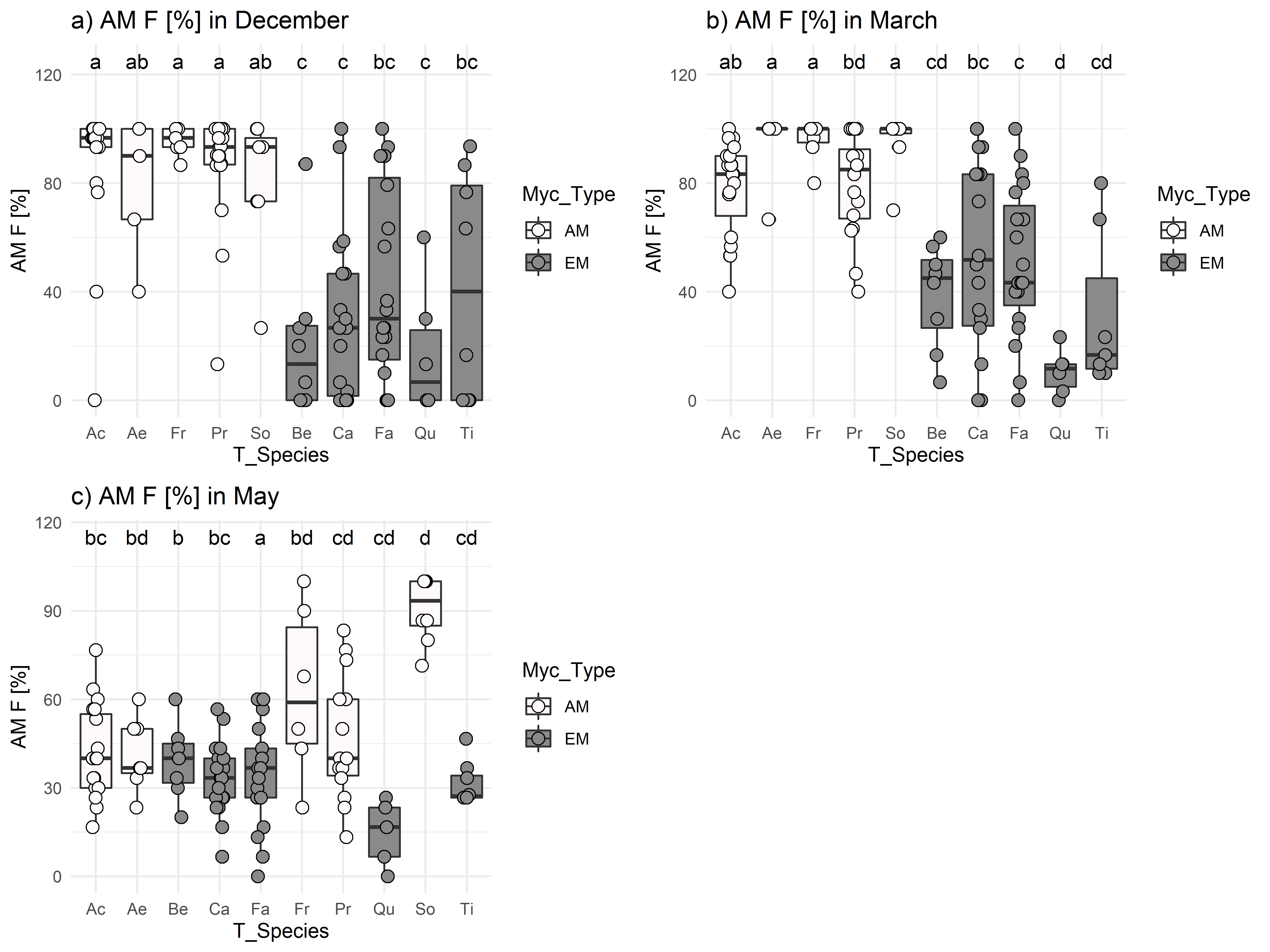

Supplement: Supplementary file 10 — Figure S7. [file ECE3-13-e10002-s006.png]

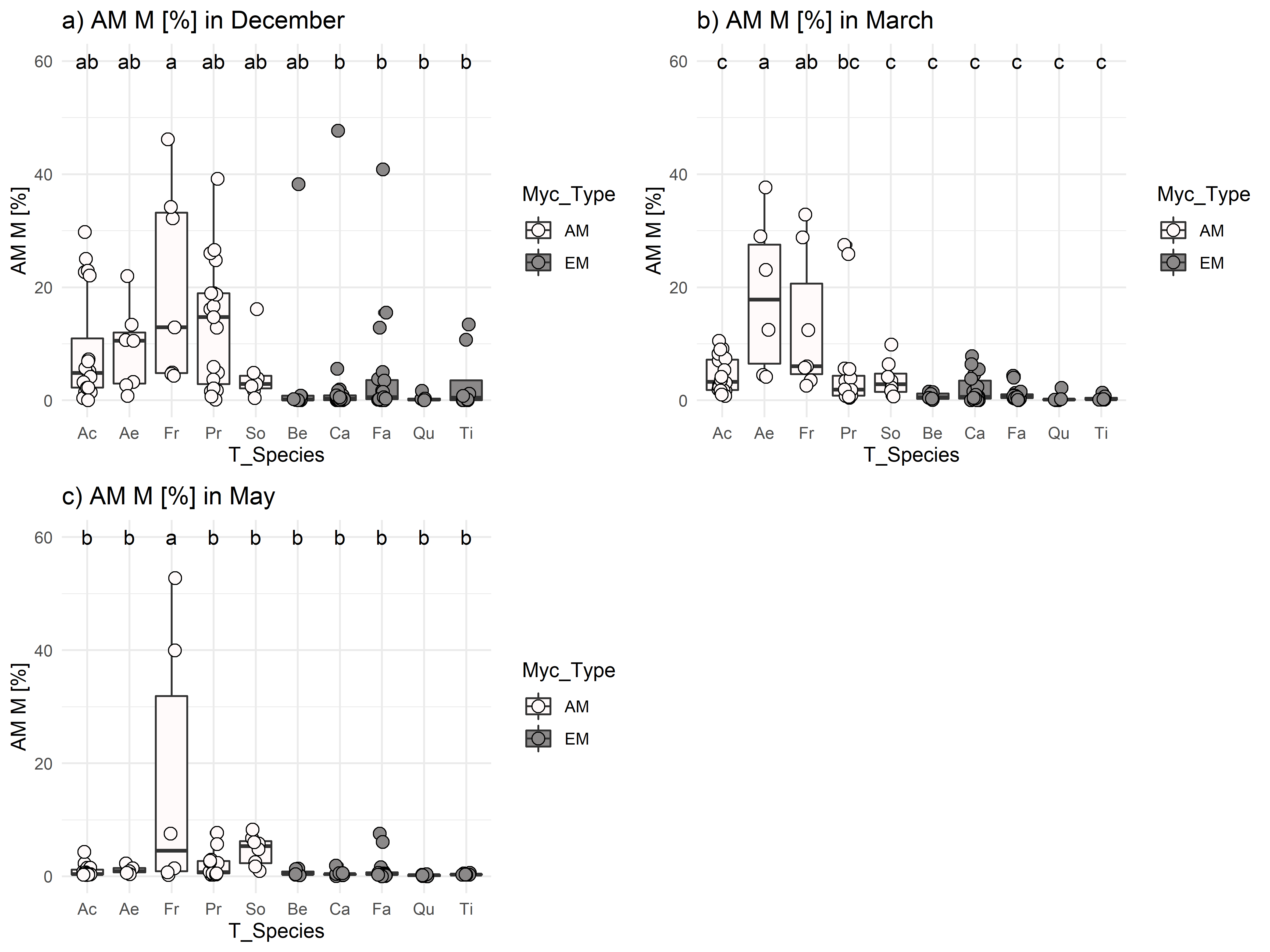

Supplement: Supplementary file 11 — Figure S8. [file ECE3-13-e10002-s014.png]

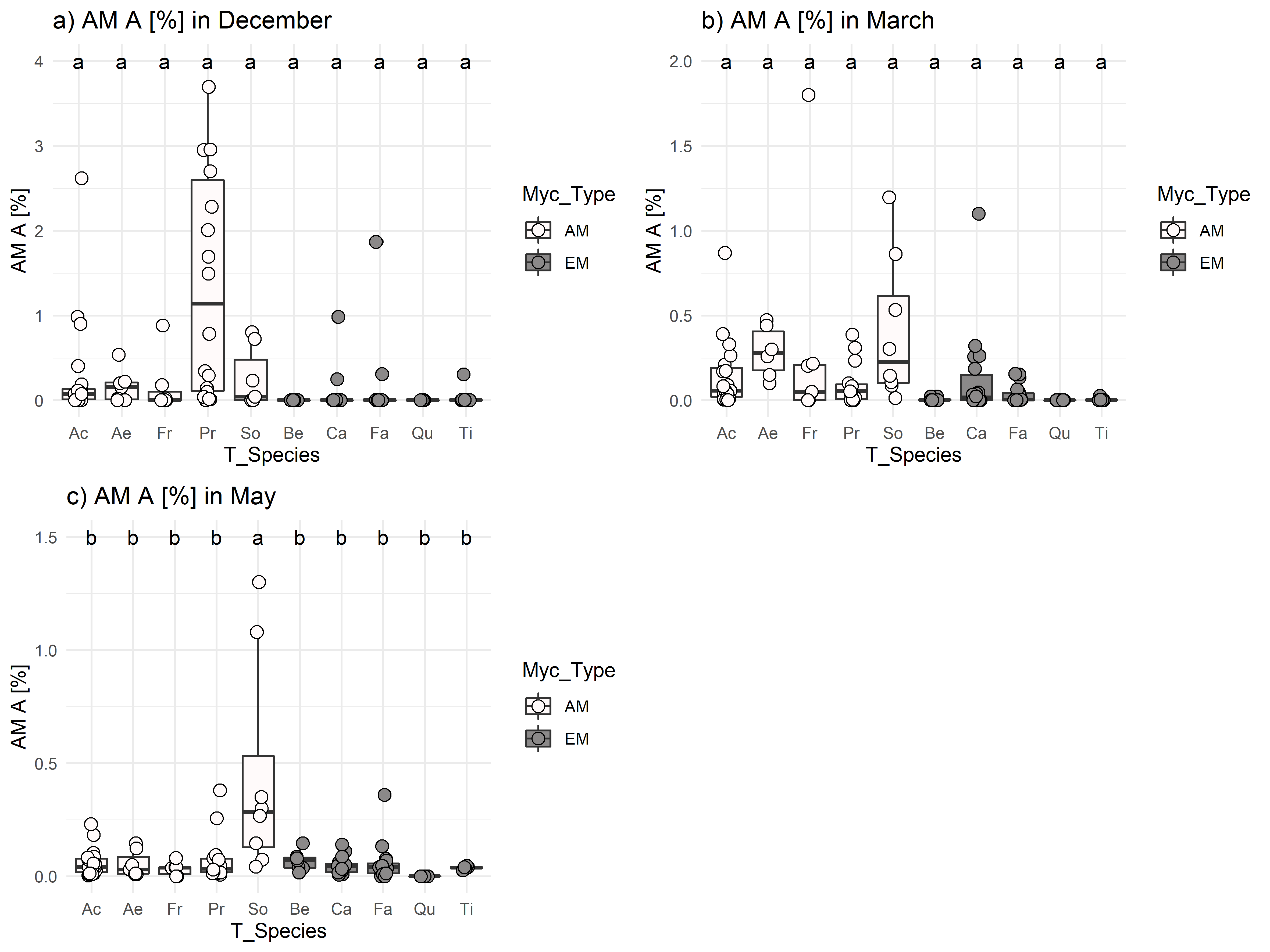

Supplement: Supplementary file 12 — Figure S9. [file ECE3-13-e10002-s010.png]
