# Supplementary material for: Temporal variation of mycorrhization rates in a tree diversity experiment
Source: Ecol Evol. 2023 Apr 19;13(4):e10002. doi: 10.1002/ece3.10002 (PMC10115898; doi:10.1002/ece3.10002)
Supplement: Supplementary file 13 — FigureCaptions. [file ECE3-13-e10002-s004.docx]

Supplemental Informations for:

Temporal variation of mycorrhization rates in a tree diversity experiment

Heike Heklau, Nicole Schindler, Nico Eisenhauer, Olga Ferlian & Helge Bruelheide

Content:

Table S1 Parameter estimates of linear models, relating EM frequency (ETC), AM frequency (AM F), intensity of the arbuscular mycorrhizal colonisation (AM M) and relative abundance of arbuscules (AM A) to sampling date (December, March, May) and tree species (T_Species). For AM F, AM M and AM A intercept is Date=December and T_Species = Ac, for ECT intercept is Date=December and T_Species = Be, Ac= *Acer pseudoplatanus*, Ae=*Aesculus hippocastanum* L., Be=*Betula pendula* Roth, Ca=*Carpinus betulus* L.*,* Fa=*Fagus sylvatica* L, Fr=*Fraxinus excelsior* L., Pr=*Prunus avium* L., Qu=*Quercus petraea* (Matt.) Liebl., So= *Sorbus aucuparia* L. and Ti=*Tilia platyphyllos* Scop. For EMT intercept is Be=*Betula pendula*.

Fig. S1 Plot map of the MyDiv experiment. The colours illustrate the selected plots and species as well as the biodiversity levels (1, 2, 4 species) and the combination of mycorrhiza types per plot: yellow = Arbuscular Mycorrhiza (AM), blue = Ectomycorrhiza (EM), green = mixing of AM and EM. Ac= *Acer pseudoplatanus*, Ae= *Aesculus hippocastanum*, Ca=*Capinus betulus*, Fa=*Fagus sylvatica*, Fr=*Fraxinus excelsior*, Pr=*Prunus avium*, Qu=*Quercus petraea*, So=*Sorbus aucuparium*, Ti=*Tilia cordata*.

Fig. S2 Fine root tips of the EMF-associated tree species, pictures taken using a stereomicroscope by Paul Kühn: a) *Betula pendula*, b) *Fagus sylvaticus,* c) *Carpinus betulus*, e) *Quercus petraea*, f) *Tilia platyphyllos*. In d) the anatomical cross section of an active root tip of *Carpinus betulus* is shown with hyphal cover around the fine root and the Hartig net between cortex cells. This picture was taken with a light microscope by Heike Heklau.

Fig. S3 Fine root tips of the tree species associated with AM, pictures taken using a stereomicroscope by Paul Kühn: a) *Aesculus hippocastanum*, c) *Prunus avium*, d) *Sorbus aucuparia*, e) *Fraxinus excelsior*. In b) the anatomical cross section of a root tip of *Aesculus hippocastanum* without hyphal cover and Hartig net. The cells of cortex are infected with endophyte fungi. This picture was taken with a light microscope by Heike Heklau.

Fig. S4 Root tips of *Acer pseudoplatanus*: a) and b), pictures taken using a stereomicroscope by Heike Heklau; c) and d) pictures taken using a scanning electron microscope by Laura Freisleben and Heike Heklau. Numerous root hairs are visible while hyphae are absent. In e) the anatomical cross section of a root tip of *Acer pseudolatanus* without hyphal cover and Hartig net and very few endophytes in the cortex cells.

Fig. S5 Mycorrhization rates by sampling date and tree species.

a) Frequency of active ectomycorrhizal root tips (ECT in %), b) frequency of arbuscular mycorrhiza (AM F in %), c) intensity of the arbuscular mycorrhizal colonisation (AM M in %) and d) relative abundance of arbuscules (AM A in %) shown by tree species: Ac= *Acer pseudoplatanus*, Ae=*Aesculus hippocastanum,* Fr=*Fraxinus excelsior*, Pr=*Prunus avium*, So=*Sorbus aucuparia* as trees predominantly associated with arbuscular mycorrhiza (AM) and Be=*Betula pendula*, Ca=*Carpinus betulus*, Fa= *Fagus sylvatica*, Qu=*Quercus petraea* and Ti=*Tilia platyphyllos* as tree species predominantly associated with ectomycorrhiza (EM).

Fig. S6 Frequency of active ectomycorrhizal root tips (ECT in %) by tree species. Small letters indicate statistically significant differences according to a Tukey post-hoc test. Tree species: Ac= *Acer pseudoplatanus*, Ae=*Aesculus hippocastanum,* Fr=*Fraxinus excelsior*, Pr=*Prunus avium*, So=*Sorbus aucuparia* as trees predominantly associated with arbuscular mycorrhiza (AM) and Be=*Betula pendula*, Ca=*Carpinus betulus*, Fa= *Fagus sylvatica*, Qu=*Quercus petraea* and Ti=*Tilia platyphyllos* as tree species predominantly associated with ectomycorrhiza (EM).

Fig. S7 Frequency of arbuscular mycorrhiza (AM F in %) by tree species. Small letters indicate statistically significant differences according to a Tukey post-hoc test. Tree species: Ac= *Acer pseudoplatanus*, Ae=*Aesculus hippocastanum,* Fr=*Fraxinus excelsior*, Pr=*Prunus avium*, So=*Sorbus aucuparia* as trees predominantly associated with arbuscular mycorrhiza (AM) and Be=*Betula pendula*, Ca=*Carpinus betulus*, Fa= *Fagus sylvatica*, Qu=*Quercus petraea* and Ti=*Tilia platyphyllos* as tree species predominantly associated with ectomycorrhiza (EM).

Fig. S8 Intensity of the arbuscular mycorrhizal colonisation (AM M in %) by tree species. Small letters indicate statistically significant differences according to a Tukey post-hoc test. Tree species: Ac= *Acer pseudoplatanus*, Ae=*Aesculus hippocastanum,* Fr=*Fraxinus excelsior*, Pr=*Prunus avium*, So=*Sorbus aucuparia* as trees predominantly associated with arbuscular mycorrhiza (AM) and Be=*Betula pendula*, Ca=*Carpinus betulus*, Fa= *Fagus sylvatica*, Qu=*Quercus petraea* and Ti=*Tilia platyphyllos* as tree species predominantly associated with ectomycorrhiza (EM).

Fig. S9 Relative abundance of arbuscules (AM A in %) by tree species. Small letters indicate statistically significant differences according to aTukey post-hoc test. Tree species: Ac= *Acer pseudoplatanus*, Ae=*Aesculus hippocastanum,* Fr=*Fraxinus excelsior*, Pr=*Prunus avium*, So=*Sorbus aucuparia* as trees predominantly associated with arbuscular mycorrhiza (AM) and Be=*Betula pendula*, Ca=*Carpinus betulus*, Fa= *Fagus sylvatica*, Qu=*Quercus petraea* and Ti=*Tilia platyphyllos* as tree species predominantly associated with ectomycorrhiza (EM).
