# Supplementary material for: Temporal variation of mycorrhization rates in a tree diversity experiment
Source: Ecol Evol. 2023 Apr 19;13(4):e10002. doi: 10.1002/ece3.10002 (PMC10115898; doi:10.1002/ece3.10002)
Supplement: Supplementary file 14 — Table S1. [file ECE3-13-e10002-s012.docx]

Table S1 Parameter estimates of linear models, relating EM frequency (ETC), AM frequency (AM F), intensity of the arbuscular mycorrhizal colonisation (AM M) and relative abundance of arbuscules (AM A) to sampling date (December, March, May) and tree species (T_Species). For AM F, AM M and AM A intercept is Date=December and T_Species = Ac, for ECT intercept is Date=December and T_Species = Be, Ac= *Acer pseudoplatanus*, Ae=*Aesculus hippocastanum* L., Be=*Betula pendula* Roth, Ca=*Carpinus betulus* L.*,* Fa=*Fagus sylvatica* L, Fr=*Fraxinus excelsior* L., Pr=*Prunus avium* L., Qu=*Quercus petraea* (Matt.) Liebl., So= *Sorbus aucuparia* L. and Ti=*Tilia platyphyllos* Scop. For EMT intercept is Be=*Betula pendula*.

|  | ECT | | AM F | | AM M | | AM A | |
| --- | --- | --- | --- | --- | --- | --- | --- | --- |
|  | Estimate | P value | Estimate | P value | Estimate | P value | Estimate | P value |
| Intercept | 41.7775 | **9.12e-07** | 87.350 | **< 2e-16** | 8.85667 | **2.6e-07** | 0.29133 | 0.2713 |
| Sampling_DateMarch | -11.0462 | 0.3108 | -9.725 | 0.185338 | -4.402 | 0.069 | -0.143 | 0.706 |
| Sampling_DateMay | -18.1424 | 0.1099 | -46.033 | **1.48e-09** | -7.9461 | **0.0011** | -0.231 | 0.542 |
| T_Species Ae |  |  | -8.101 | 0.493804 | 0.179 | 0.957 | -0.130 | 0.802 |
| T_Species Be |  |  | -66.046 | **1.39e-10** | -3.841 | 0.223 | 0.922 | 0.063 |
| T_Species Ca | 6.4946 | 0.5151 | -56.993 | **3.55e-12** | -5.666 | **0.0193** | -0.226 | 0.5519 |
| T_Species Fa | 0.2736 | 0.9783 | 44.401 | **2.12e-08** | -4.470 | 0.061 | 0.545 | 0.146 |
| T_Species Fr | 5.0846 | 0.6753 | 8.364 | 0.422255 | 11.048 | **0.001** | -0.136 | 0.793 |
| T_Species Pr | 2.4704 | 0.8056 | -0.402 | 0.958549 | 4.523 | 0.062 | 158.788 | **3.59e-05** |
| T_Species Qu | 3.8340 | 0.7621 | 70.128 | **7.60e-10** | -8.484 | **0.016** | -0.291 | 0.597 |
| T_Species So | -3.7554 | 0.7487 | -7.240 | 0.487120 | -4.233 | 0.201 | -0.034 | 0.948 |
| T_Species Ti | -7.4398 | 0.5258 | -45.240 | **7.55e-06** | -5.575 | 0.078 | -0.251 | 0.611 |
| Sampling_DateMarch:T_SpeciesAe |  |  | 24.842 | 0.114379 | 13.846 | **0.0044** | 0.269 | 0.723 |
| Sampling_DateMay:T_SpeciesAe |  |  | 8.213 | 0.591928 | 0.040 | 0.993 | 0.125 | 0.8656 |
| Sampling_DateMarch:T_SpeciesBe |  |  | 27.171 | **0.046300** | 0.099 | 0.982 | -106.491 | 0.130 |
| Sampling_DateMay:T_SpeciesBe |  |  | 63.881 | 7.11e-06 | 3.550 | 0.439 | -0.914 | 0.205 |
| Sampling_DateMarch:T_SpeciesCa | 5.3781 | 0.6776 | 32.705 | **0.002060** | 3.196 | 0.356 | 0.204 | 0.707 |
| Sampling_DateMay:T_SpeciesCa | -2.6169 | 0.8446 | 49.211 | **3.95e-06** | 5.203 | 0.130 | 0.212 | 0.694 |
| Sampling_DateMarch:T_SpeciesFa | 4.4050 | 0.7334 | 17.512 | 0.092054 | 1.026 | 0.764 | -0.660 | 0.219 |
| Sampling_DateMay:T_SpeciesFa | 3.6099 | 0.7894 | 36.942 | 0.000525 | 4.750 | 0.171 | -0.548 | 0.314 |
| Sampling_DateMarch:T_SpeciesFr | -30.4826 | 0.0569 | 9.725 | 0.495188 | -2.365 | 0.614 | 0.312 | 0.672 |
| Sampling_DateMay:T_SpeciesFr | 13.5993 | 0.4134 | 12.799 | 0.384424 | 5.144 | 0.288 | 0.109 | 0.886 |
| Sampling_DateMarch:T_SpeciesPr | 16.7926 | 0.2010 | 2.478 | 0.816596 | -3.688 | 0.294 | -137.882 | **0.013** |
| Sampling_DateMay:T_SpeciesPr | 10.1604 | 0.4552 | 5.563 | 0.610458 | -3.443 | 0.337 | -157.002 | **0.006** |
| Sampling_DateMarch:T_SpeciesQu | -16.5749 | 0.3194 | 3.058 | 0.839502 | 4.480 | 0.368 | 0.143 | 0.855 |
| Sampling_DateMay:T_SpeciesQu | 16.6234 | 0.3262 | 43.281 | **0.006201** | 7.747 | 0.134 | 0.231 | 0.775 |
| Sampling_DateMarch:T_SpeciesSo | 30.5378 | **0.0484** | 25.031 | 0.073427 | 3.450 | 0.452 | 0.291 | 0.686 |
| Sampling_DateMay:T_SpeciesSo | 18.2717 | 0.2453 | 56.518 | **6.60e-05** | 7.924 | 0.085 | 0.419 | 0.561 |
| Sampling_DateMarch:T_SpeciesTi | 32.3592 | **0.0366** | -0.643 | 0.963203 | 1.511 | 0.742 | 0.108 | 0.881 |
| Sampling_DateMay:T_SpeciesTi | 3.0956 | 0.8437 | 35.288 | **0.009860** | 5.012 | 0.263 | 0.229 | 0.744 |
